# Catecholaminergic Innervation of Central and Peripheral Auditory Circuitry Varies with Reproductive State in Female Midshipman Fish, *Porichthys notatus*

**DOI:** 10.1371/journal.pone.0121914

**Published:** 2015-04-07

**Authors:** Paul M. Forlano, Zachary N. Ghahramani, Camillia M. Monestime, Philip Kurochkin, Alena Chernenko, Dmitriy Milkis

**Affiliations:** 1 Department of Biology, Brooklyn College, City University of New York, Brooklyn, NY, United States of America; 2 Program in Neuroscience, City University of New York, New York, NY, United States of America; 3 Program in Ecology, Evolutionary Biology and Behavior, City University of New York, New York, NY, United States of America; 4 Program in Behavioral and Cognitive Neuroscience, The Graduate Center, City University of New York, New York, NY, United States of America; 5 Aquatic Research and Environmental Assessment Center, Brooklyn College, Brooklyn, NY, United States of America; Claremont Colleges, UNITED STATES

## Abstract

In seasonal breeding vertebrates, hormone regulation of catecholamines, which include dopamine and noradrenaline, may function, in part, to modulate behavioral responses to conspecific vocalizations. However, natural seasonal changes in catecholamine innervation of auditory nuclei is largely unexplored, especially in the peripheral auditory system, where encoding of social acoustic stimuli is initiated. The plainfin midshipman fish, *Porichthys notatus*, has proven to be an excellent model to explore mechanisms underlying seasonal peripheral auditory plasticity related to reproductive social behavior. Recently, we demonstrated robust catecholaminergic (CA) innervation throughout the auditory system in midshipman. Most notably, dopaminergic neurons in the diencephalon have widespread projections to auditory circuitry including direct innervation of the saccule, the main endorgan of hearing, and the cholinergic octavolateralis efferent nucleus (OE) which also projects to the inner ear. Here, we tested the hypothesis that gravid, reproductive summer females show differential CA innervation of the auditory system compared to non-reproductive winter females. We utilized quantitative immunofluorescence to measure tyrosine hydroxylase immunoreactive (TH-ir) fiber density throughout central auditory nuclei and the sensory epithelium of the saccule. Reproductive females exhibited greater density of TH-ir innervation in two forebrain areas including the auditory thalamus and greater density of TH-ir on somata and dendrites of the OE. In contrast, non-reproductive females had greater numbers of TH-ir terminals in the saccule and greater TH-ir fiber density in a region of the auditory hindbrain as well as greater numbers of TH-ir neurons in the preoptic area. These data provide evidence that catecholamines may function, in part, to seasonally modulate the sensitivity of the inner ear and, in turn, the appropriate behavioral response to reproductive acoustic signals.

## Introduction

Catecholamines are well documented as important regulators of reward and motivated social behavior, but are also known modulators of sensory and motor systems [1–3]. Recent studies, largely in songbirds, provide strong evidence that both dopamine and noradrenaline are important neuromodulators of vocal-acoustic communication, especially in the context of appropriate brain and behavioral response to social auditory signals (for review see [4]). For seasonal breeding vertebrates, hormone regulation of catecholaminergic (CA) innervation of sensory areas may be a conserved mechanism for differential seasonal response to conspecific vocalizations [5, 6]. In support of this, mimicking seasonal changes in circulating steroid levels has provided evidence that estrogen increases CA innervation of midbrain and forebrain auditory nuclei in female white-throated sparrows as measured by changes in tyrosine hydroxylase immunoreactivity (TH-ir) [7, 8]. Natural seasonal changes in TH-ir innervation of auditory nuclei that reflect differences in reproductive state (i.e., breeding vs. non-breeding) is largely unexplored, and no studies have investigated reproductive state (or hormone)-induced plasticity of CA innervation of the auditory periphery and hindbrain auditory processing areas.

The plainfin midshipman fish, *Porichthys notatus*, has proven to be an excellent model to explore mechanisms underlying seasonal plasticity in audition related to reproductive social behavior [9–11]. Midshipman spend roughly nine months of the year in deep waters off the coast of northern California and the Pacific Northwest and migrate into the rocky intertidal zone to spawn in late May to mid-August. Males excavate nests under rocks, defend these territories from neighboring males, and court females at night by a long duration advertisement call (“hum”) [10, 12]. Females find nesting males by localizing the source of the hum, deposit their eggs in a single nest and head back offshore while the male cares for the young and continues to court other females until the nest is full of developing embryos [10, 12, 13].

Female and male midshipman undergo a seasonal change in auditory frequency sensitivity at the level of the inner ear such that summer, reproductive fish can better encode higher harmonic frequencies of the hum compared to non-reproductive winter fish [14–16], and this auditory plasticity can be induced by implanting non-reproductive females with either testosterone or estradiol [17] which raises circulating T/ E2 to their natural peak seen during the pre-spawning period [18]. More recent studies have also shown that summer females have a significantly greater number of hair cells as well as an increase in number of large-conductance, calcium-activated potassium (BK) channel transcript subunits in the saccule, the main endorgan of hearing, compared to winter females [19, 20]. Indeed, pharmacological blockade of BK channel function mimics seasonal plasticity in auditory encoding [19, 20]. These structural-type changes at the auditory periphery occur over several weeks, likely driven by hormone- mediated changes in gene expression. Seasonal plasticity in the central auditory system of midshipman has not been investigated and may include neuromodulators which mediate a drastic change in auditory-driven female behavior that occurs *within* the reproductive season: a directed behavioral response to an underwater playback of the fundamental frequency of male advertisement call (phonotaxis) occurs in females that are gravid (full of eggs), but is entirely absent when spent (void of eggs), immediately post-spawning, after having released most of their eggs [9, 13, 21, 22]. Catecholamines are good candidates for this function, as they are thought to modulate the salience of socially relevant stimuli and mediate transitions between various behaviors (see [6, 23]).

**Fig 1:**
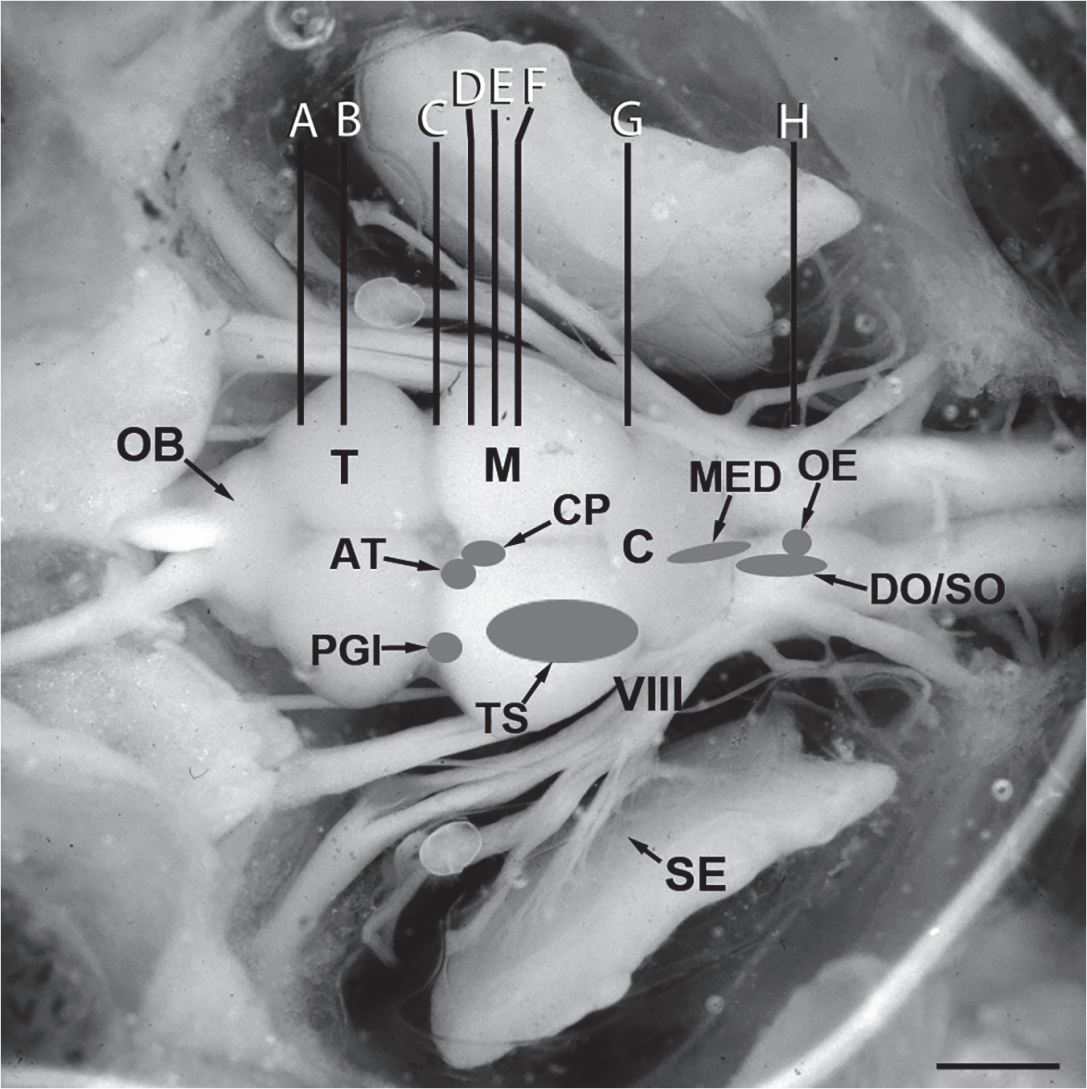
Dorsal view of an exposed midshipman brain and inner ear. Shaded areas indicate location of auditory nuclei analyzed in this study (adapted from [25]). Vertical lines (A-H) indicate levels of transverse sections seen in Fig 2. Abbreviations: AT, anterior tuberal nucleus; C, cerebellum; CP, central posterior nucleus of the thalamus; DO, descending octaval nucleus; M, midbrain; MED, medial octavolateralis nucleus; OB, olfactory bulb; OE, octavolateralis efferent nucleus; PGl, lateral division of nucleus preglomerulosus; SE, saccular epithelium of the inner ear; SO, secondary octaval nucleus; T, telencephalon; TS, torus semicircularis; VIII, eighth nerve. Scale bar = 1.5 mm.

**Fig 2:**
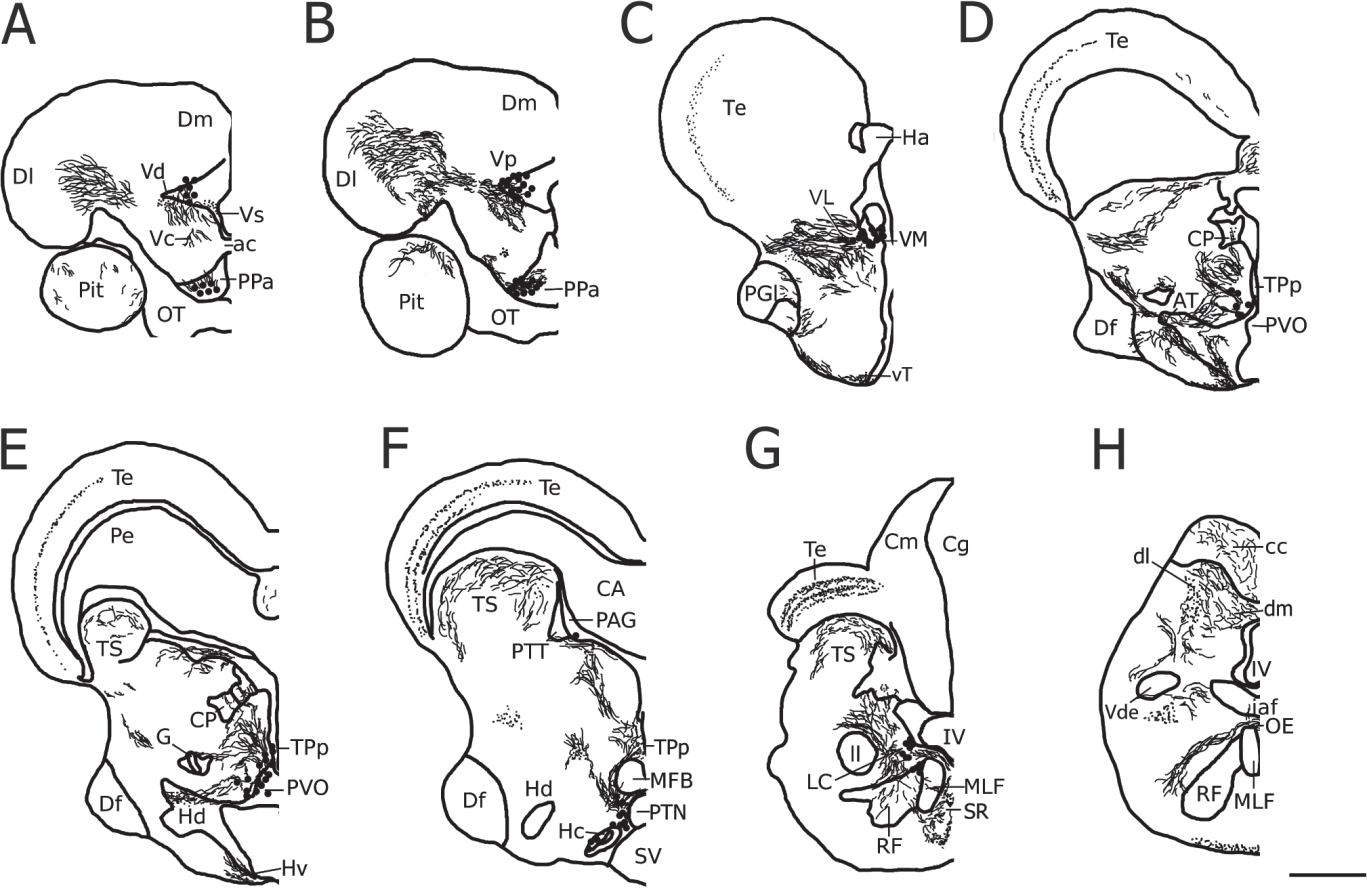
A representative series of line drawings illustrating rostro-caudal distribution of major tyrosine hydroxylase immunoreactive (TH-ir) cell populations (large dots), fibers (lines) and terminals (small dots) in the midshipman brain covering areas analyzed in the current study (adapted from [24]). Abbreviations: ac, anterior commissure; AT, anterior tuberal nucleus; CA, cerebral aqueduct; cc, cerebellar crest; Cm, molecular layer of the corpus of the cerebellum; Cg, granular layer of the corpus of the cerebellum; CP, central posterior nucleus of the thalamus; Df, diffuse nucleus of the hypothalamus; dl, dorsolateral division of the descending octaval nucleus; Dl, lateral zone of area dorsalis of the telencephalon; dm, dorsomedial division of the descending octaval nucleus; Dm, medial zone of area dorsalis of the telencephalon; G, nucleus glomerulosus; Ha, habenula; Hc, central periventricular hypothalamus; Hd, dorsal periventricular hypothalamus; Hv, ventral periventricular hypothalamus; iaf, internal arcuate fiber tract; IV, fourth ventricle; LC, locus coeruleus; ll, lateral lemniscus; MED, medial octavolateralis nucleus; MFB, medial forebrain bundle; MLF, medial longitudinal fasciculus; OB, olfactory bulb; OE, octavolateralis efferent nucleus; OT, optic tract; PAG, periaqueductal gray; Pe, periventricular cell layer of the torus semicircularis; PPa, anterior parvocellular preoptic nucleus; PGl, lateral division of nucleus preglomerulosus; Pit, pituitary; PTN, posterior tuberal nucleus; PTT, paratoral tegmentum; PVO, paraventricular organ; RF, reticular formation; SR, superior raphe; SV, saccus vasculosus; Te, midbrain tectum; TPp, periventricular posterior tuberculum; TS, torus semicircularis; Vc, central nucleus of area ventralis of the telencephalon; Vd, dorsal nucleus of area ventralis of the telencephalon; Vde, descending tract of the trigeminal nerve; VL, ventrolateral nucleus of the thalamus; VM, ventromedial nucleus of the thalamus; Vp, postcommissural nucleus of area ventralis of the telencephalon; Vs, supracommissural nucleus of area ventralis of the telencephalon; vT, ventral tuberal hypothalamus. Scale bar = 500 μm.

The auditory system in midshipman is well characterized anatomically (Fig 1) and we recently demonstrated robust CA innervation throughout all levels of central (Fig 2) and peripheral auditory circuitry in midshipman [24]. Like other teleosts, primary afferents from the saccule synapse onto hindbrain neurons of the descending octaval nucleus (DO) which are connected to secondary octaval neurons (SO); both hindbrain groups project to the midbrain torus semicircularis (TS), which in turn projects to several diencephalic nuclei including the anterior tuberal nucleus of the hypothalamus (AT), lateral division of nucleus preglomerulosus (PGl) and the central posterior nucleus of the thalamus (CP) which relays information to telencephalic nuclei [25–29]. Furthermore, the hindbrain octavolateralis efferent nucleus (OE) projects to the inner ear endorgans and lateral line system [25, 26, 30–32]. Interestingly, TH-ir neurons in the periventricular posterior tuberculum (TPp), proposed homologues of A11 dopaminergic (DA) neurons in tetrapods ([33–35]; but see [36]), send projections directly to the saccule and also appear to innervate the OE, DO and CP as well as the periaqueductal gray (PAG) and spinal cord ([24]; for zebrafish see [34, 37]). Furthermore, TPp neurons receive auditory input indirectly via the PAG (see [24, 38]). In addition, TH-ir TPp neurons send ascending projections to the ventral telencephalon ([24]; for zebrafish see [34, 39]) and are therefore in a key position to modulate sensory-motor integration in the context of social auditory stimuli. In support of this, we showed that males exposed to advertisement calls of other males exhibit a cFos response (proxy for neural activation) in TPp TH-ir neurons which are thus active in response to social acoustic cues [40].

Here we tested the hypothesis that female midshipman show seasonal changes in CA innervation of auditory nuclei. This plasticity may serve to modulate auditory sensitivity which is known to vary with reproductive state and is adaptive to better encode the male advertisement call during the breeding season [10,11]. In order to compare wild-caught gravid, reproductive summer females and non-reproductive winter females, we utilized quantitative fluorescent immunohistochemistry to measure TH-ir fiber density in six auditory nuclei that span the forebrain, midbrain and hindbrain (Fig 1) (see [24–26]). In addition, we analyzed TH-ir innervation of somata and dendrites of the cholinergic OE [41] by double-labeling with a choline acetyltransferase (ChAT) antibody, and quantified both TH-ir and ChAT-ir putative terminals on the sensory epithelium of the saccule of the inner ear. Finally, we counted numbers of TH-ir neurons in TPp and locus coeruleus (LC) which are known to contribute widespread dopaminergic (DA) and noradrenergic (NA) input, respectively, to auditory nuclei [24, 42], as well as TH-ir neurons in hypothalamic AT (above) and the anterior parvocellular preoptic nucleus (PPa), the latter which is known to be involved in regulation of the reproductive axis in teleost fishes [43]. We report seasonal differences in CA fiber innervation of several areas in the midshipman auditory pathway which supports the hypothesis that catecholamines play an important role in seasonal auditory-driven social and reproductive behavior.

## Methods

### Ethics Statement

All experimental animal procedures performed in this study were approved by the Institute for Animal Care and Use Committee of the University of California, Davis (Protocol Number: 15977), University of Washington (Protocol Number: 4079–01) and CUNY Brooklyn College (Protocol Number: 260). Animals were collected from the field under Department of Fish and Game Permit 802021–01 (California) and 13–161 (Washington).

### Animals

Seasonal time periods are distinguished by multiple characters including fluctuations in circulating steroid hormone levels and gonadosomatic index (GSI, ratio of gonad weight to body weight), different habitats (offshore sites versus shallow intertidal zone) and vocal and spawning behaviors (see introduction and [10, 18]). In general, the non-reproductive period is defined by fish found at the deepest collection depths offshore between December and February, and which have low GSI and basal levels of circulating steroids. The pre-nesting period, not measured in the present study, occurs in the late winter and spring when fish are collected at the shallowest depths offshore and have the greatest variation in GSI and circulating gonadal steroids which reflects the period of gonadal recrudescence. The nesting period occurs in mid-May to August when females are collected from nests in the intertidal zone at low tide and are gravid with large yolked ova.

Female midshipman were collected by hand from nests at low tide during the reproductive season (June) in Tomales Bay, adjacent to the town of Marshall, CA and held in the Bodega Marine Lab until sacrificed within 24 hrs of capture. Only gravid females with a GSI above 10% were used (n = 6). Non-reproductive animals were collected by otter trawl (R/V Kittiwake) in December in Puget Sound off the Port of Edmonds, WA and shipped to Brooklyn College where they were sacrificed within 6 days of collection (n = 6). All non-reproductive females were confirmed to have completely regressed ovaries (below). A previous study demonstrated that physiological changes in the peripheral auditory system of summer gravid females occur after > 25 d in captivity while those recorded within 15 days of collection show consistent peripheral auditory encoding seen in the reproductive period [16]. Thus, all animals were sacrificed within a time period that should accurately reflect their respective reproductive state. Importantly, midshipman from both populations on the west coast have been well studied; they exhibit the same life history as described above, and show identical seasonal changes in auditory frequency encoding [14–16].

### Tissue Collection and Immunohistochemistry

All animals were deeply anesthetized in 0.025% benzocaine (Sigma Chemicals, St Louis, MO) in seawater, measured, weighed, and then transcardially perfused with ice cold teleost ringers followed by 4% paraformaldehyde in 0.1 M of phosphate buffer (PB, pH 7.2). Brains and saccules were removed and post-fixed for 1 hour in the same fixative, rinsed and stored in PB until transferred to a 30% sucrose PB solution about 24–48 hours prior to cryosectioning. Brains were sectioned at 25 μm in the transverse plane and collected in 2 series on subbed slides. One saccular epithelium was dissected off its otolith, sectioned at 20 μm in the transverse plane and collected into two series. Slides were stored at -80°C until all tissue was collected and sectioned. All brain tissue and all saccules from both groups, respectively, were processed simultaneously for immunohistochemistry.

Fluorescence immunohistochemistry was modified from a previous protocol [24]. Briefly, after slides were allowed to warm to room temperature, they were rinsed in 0.1M phosphate buffered saline (PBS, pH 7.2) 2x 10min, followed by a 1 hour soak in a blocking solution of 0.1M PBS + 10% donkey serum (DS) + 0.3% Triton X-100. Primary antibodies, monoclonal mouse anti-TH (1:1000; Millipore, Billerica, MA, #MAB318) and polyclonal goat anti-ChAT (1:200; Millipore, #AB144), were diluted and mixed in the above blocking solution and incubated on sections in a humidified chamber at room temperature for 16 hrs followed by 5x 10 min rinses in PBS + 0.5% DS, then incubated 2 hr with anti-mouse AlexaFlour 568 and anti-goat AlexaFlour 488 (1:200; Molecular Probes, Life Technologies), washed 4 x 10 min in PBS and coverslipped with Prolong Gold containing DAPI nuclear stain (Life Technologies). Slides were allowed to cure in the dark at room temperature for approximately 48 hours, then subsequently sealed with nail polish and stored at 4°C.

### Image Acquisition and Anatomy

Brain images were acquired on an Olympus BX61 epifluorescence compound microscope (Tokyo, Japan) using MetaMorph imaging and processing software (Molecular Devices, Sunnyvale, CA). Each nucleus analyzed was imaged with a 20x objective at the same exposure time and light level. Each photomicrograph was taken consecutively using Texas Red, GFP (where appropriate) and DAPI filter sets (Chroma, Bellow Falls, VT). Imaging at this magnification (relatively shallow depth of field) made it necessary to capture images in a z-stack (sequential acquisition of a specified area with the focused position varied by a constant step ranging around a central plane) that contained between 5–9 levels, each with a thickness of 1μm. These photomicrographs were combined into a single projected image in MetaMorph permitting crisp, confocal-like images for quantitative analyses. Nuclear boundaries were drawn post-acquisition using DAPI to define cytoarchitecture. Sampling strategy was determined per region to account for intrinsic variation in size between nuclei. In the case of tissue loss or damage, the opposite side of the brain was used (for unilateral sampling), or the section was omitted. An animal was excluded from analysis for a particular brain area if section damage resulted in more than half the average number of sections sampled per animal to be unusable. In order to understand the overall effect of season on TH-ir density within central auditory circuitry, a two-way ANOVA was utilized with region of interest and season as factors. A series of *a priori* independent samples *t*-tests were performed to compare TH-ir fiber density and/or TH-ir cell number in each region of interest between summer and winter animals. All statistics are reported herein as mean ± standard error (SE), unless otherwise noted. Experimenter was blind to treatment conditions of all slides analyzed.

#### Cell Counts

Manual quantification of TH-ir neurons was performed in the LC, TPp, PPa and AT (see below). Individual TH-ir cells were counted only if they contained a clear nucleus which was colocalized with DAPI. In order to standardize variation in the number of sections collected in each brain region of interest between groups, counts of absolute TH-ir cell numbers were adjusted to the average number of TH-ir neurons per section. The LC was identified by the presence of TH-ir cells dorsolateral to the medial longitudinal fasciculus (MLF) in the isthmal region between the hindbrain and midbrain. Sampling began in the caudal LC with the bilateral appearance of TH-ir cells, continuing serially in the caudal-to-rostral direction for an average of 9.6 (± 1.8 SD) consecutive sections per animal until their disappearance. Each photomicrograph was comprised of a z-stack of 9 levels taken of TH-ir neurons unilaterally. An unpaired two-tailed *t*-test determined that there was no difference in the number of sections between groups (p > 0.05).

Sampling of the TPp began caudally with the appearance of dense clusters of large, pear-shaped TH-ir cells medial to the medial forebrain bundle (MFB) that extended ventrolaterally along the lateral border of the paraventricular organ (PVO). Using a z-stack of 9 levels, up to three 20x photomicrographs were needed to capture all TH-ir cells per section and photomicrographs were aligned prior to analysis to avoid overlap. The TPp was analyzed serially for an average of 6 (± 3 SD) consecutive sections in the caudal-to-rostral direction until the disappearance of large, pear-shaped TH-ir cells adjacent to the midline. The area of TH-ir cells and proximal dendrites were also quantified in the TPp by thresholding the cells and processes (see below) and measuring the area of signal covering a defined field of view (143,139 μm^2^) of each image capturing the TPp. An unpaired two-tailed *t*-test determined that there was no difference in the number of sections between groups (p > 0.05).

Sampling of the PPa began rostral to the horizontal commissure, with the appearance of parvocellular TH-ir cells clustered ventrolateral to the midline in the caudal-most aspect of the nucleus and continued serially in the rostral direction until their disappearance. A single photomicrograph comprised of a z-stack of 9 levels was taken unilaterally of TH-ir neurons for an average of 10.8 (± 2.9 SD) consecutive sections per animal. An unpaired two-tailed *t*-test determined that there was no difference in the number of sections between groups (p > 0.05).

#### Fiber Analysis

TH-ir fiber innervation of auditory nuclei was segmented using the “Inclusive Threshold” feature of MetaMorph. Due to subtle variation in background staining from section to section, the threshold for each image was set manually. Threshold levels were determined so that pixels selected by MetaMorph were in accordance with what the blind observer deemed to be TH-ir (e.g., unambiguous labeling clearly above unlabeled structures in the same image). This method is in line with previously published work examining plasticity of TH-ir fibers in songbirds [7,8]. To obtain an average TH-ir fiber density for each nucleus, the area of signal covering the area defined as the nucleus of interest was measured across several consecutive serial sections (see below). Nuclear boundaries as defined by DAPI were traced and overlaid onto the Texas Red channel where TH-ir fiber density, integrated (total) intensity and average intensity were quantified using the Region Statistics feature in MetaMorph. In some cases where more diffuse nuclei were analyzed (SO, DO), the field of view was positioned according to neighboring landmarks to encompass the nucleus of interest (see below; [40]).

Auditory hindbrain nuclei that were analyzed included a region spanning laterally within and between the dorsolateral division of DO (DOdl) and the medial octavolateralis nucleus (MED). Although MED receives lateral line input, it is interconnected with DOdl (see Discussion). Sampling of DOdl+MED began dorsolateral to the fourth ventricle (IV) just rostral to the level of OE. A single photomicrograph per section was taken unilaterally, with the midline and IV serving as the medial boundary, and the lateral boundary defined by DOdl lying adjacent to the central tract of the lateral line nerve (see [25, 44]). Sampling continued in the caudal-to-rostral direction, including the appearance of the MED lying adjacent to IV, for an average of 5.7 (± 1.9 SD) sections per animal using a z-stack of 7 levels. TH-ir fibers were analyzed within the boundaries of each image (approximately 143,139 μm^2^). An unpaired two-tailed *t*-test determined that there was no difference in the number of sections between groups (p > 0.05).

Sampling of the ventral division of SO (SOv) began just rostral to OE. A single, unilateral photomicrograph was taken of SOv, with the rostral intermediate DO acting as the dorsolateral boundary of the image (see [25, 40]) and TH-ir fibers were analyzed within the field of view of each image (approximately 143,139 μm^2^). Sampling continued in the caudal-to-rostral direction for an average of 4.3 (± 1.4 SD) sections per animal using a z-stack of 7 levels. An unpaired two-tailed *t*-test determined that there was no difference in the number of sections between groups (p > 0.05).

The OE has two distinct divisions, caudal (OEc) and rostral (OEr), which contain large, cholinergic (ChAT-ir) neurons clustered on the midline just ventral to IV [41]. Robust TH-ir fibers make putative contacts with ChAT-ir somata and dendrites [24]. Sampling of the OE began in the caudal division and continued in the rostral direction and subsequent OEr sections containing ChAT-ir cells were sampled. ChAT-ir dendrites positioned laterally to the somata in OE were also included in the analysis, making it necessary to take up to three 20x photomicrographs per section to capture all regions of interest. Boundaries were drawn around clusters of ChAT-ir somata in the center of the OE, as well as the laterally positioned ChAT-ir dendritic processes. Along with TH-ir density measurements, a colocalization analysis was performed to determine the percentage of ChAT-ir area of OE somata and dendrites contacted by TH-ir fibers. To do this, both TH-ir and ChAT-ir signal were thresholded (as above), and colocalization measurements (area of overlap between the two fluorescent probes) were made using traced regions defined by the ChAT-ir signal. On average, 5.6 (± 2.2 SD) sections per animal were used for ChAT-ir somata, and 6.7 (± 2.8 SD) sections per animal were used for ChAT-ir dendrites captured using a z-stack of 7 levels. An unpaired two-tailed *t*-test determined that there was no difference in the number of sections between groups (p > 0.05, in both cases).

Sampling of the TS began at the level of the valvula in caudal midbrain. Since the entire region of interest would not fit into a single 20x photomicrograph, two adjacent images were taken at each level sampled to encompass the entire nucleus. A boundary was drawn around the medial and lateral extent of the TS across the two images. TH-ir fibers were analyzed within the boundaries of each image and data were combined into a single density measurement. Laterally, landmarks were used to assure that there was no overlap in photomicrographs or boundary tracings. Moving in the caudal-to-rostral direction, every fourth section was sampled unilaterally until the appearance of CP. An average of 6.1 (± 1.2 SD) sections was analyzed per animal using a z-stack of 8 levels, and an unpaired two-tailed *t*-test determined that there was no difference in the number of sections between groups (p > 0.05).

Sampling of the compact division of the CP (CPc) began with the DAPI-labeled wing-shaped band of cells lateral to the midline. A boundary was drawn around the CPc, and a TH-ir fiber density analysis was performed within. CPc was sampled unilaterally across 3 consecutive sections in the caudal-to-rostral direction using a z-stack of 8 levels, and an unpaired two-tailed *t*-test determined that there was no difference in the number of sections between groups (p > 0.05).

Similar to the TS, PGl was sampled by two adjacent images at each level to encompass the entire nucleus. A boundary defined by a thick band of DAPI cells was drawn around the dorsomedial and ventrolateral extent of the PGl across the two images to avoid including PGm (non-auditory) in the analysis. TH-ir fibers were analyzed within the boundaries of each image and data were combined into a single density measurement. Laterally, landmarks were used to assure that there was no overlap in photomicrographs or boundary tracings. Sampling began at the level of the horizontal commissure, and, moving in the caudal-to-rostral direction, every other section was sampled unilaterally for an average of 6.1 (± 1 SD) sections per animal using a z-stack of 9 levels. An unpaired two-tailed *t*-test determined that there was no difference in the number of sections between groups (p > 0.05).

The AT is located in the ventral hypothalamus, rostral to the dorsal periventricular hypothalamus and dorsal to the lateral hypothalamus. An average of 4.6 (± 1.9 SD) consecutive sections were imaged unilaterally in the caudal-to-rostral direction using a z-stack of 5 levels. A boundary around AT was drawn in the DAPI channel and transferred to the Texas Red channel where TH-ir fiber innervation was quantified. TH-ir cells identified in the lateral-most aspect of the nucleus were also counted for analysis. An independent samples *t*-test determined that there was a difference in the number of sections between groups (p = 0.0126).

### Analysis of the Saccular Epithelium

Immunohistochemistry was identical as above for brain tissue except TH-ir was visualized with anti-mouse AlexaFluor 680 and ChAT-ir with anti-goat AlexaFluor 568, respectively. Saccular epithelia from summer and winter females were imaged on an Olympus Fluoview FV10i confocal microscope (Olympus, Center Valley, PA). Two sections, one section apart, were selected approximately every six sections for an average of 9 sections (± 2.6 SD) to sample throughout the sensory epithelium. Three images, left, middle and right, were acquired from each section for an average of 27 images (± 7.8 SD) sampled per animal. To prevent redundancies in analysis, images acquired from adjacent areas were checked for overlapping cellular structures or signal. An independent samples *t*-test determined that there was no difference in the number of sections or number of images sampled between groups (p > 0.05).

Using the Fluoview FV10i-SW image acquisition software, each area was acquired using a 7μm z-stack with a 0.5μm step size. All images were taken using a 60x oil-immersion objective and 2x digital zoom for a magnification of 120x at a resolution of 512 x 512 pixels. Each image was taken consecutively in three channels using different excitation wavelengths: DAPI (359 nm), Alexa Fluor 568 (577 nm), and Cy5.5 (673 nm).

To analyze TH-ir and ChAT-ir puncta in the saccular epithelium, raw image stacks of each channel were combined in MetaMorph and tissue borders in the field of view were traced for density measurements. TH-ir and ChAT-ir signal from each channel was then thresholded (as above) and the number and size of puncta per section was quantified using the integrated morphometry analysis (IMA) feature of MetaMorph. In order to exclude thick, longitudinal TH-ir and ChAT-ir fiber tracts from the puncta analysis, size and shape filters were applied so that objects of interest between 4–150 pixels in size with a shape factor of ≥0.6 (with a value of 1.0 representing a perfect circle) were quantified.

## Results

### Morphometrics

As expected, summer, reproductive females captured from intertidal nests had a much greater ovary to body weight ratio (mean GSI = 29.12%; range = 20.9–41.6%) compared to non-reproductive females (mean GSI = 0.98%; range = 0.9–1.1%) collected from deep offshore waters in the winter. Average body size measured by standard length (SL) was largely overlapping between seasons (mean summer SL = 12.27 cm, range = 11.4–13 cm; mean winter SL = 11.2 cm, range = 10.10–12.2 cm), but summer females were approximately 9.1% larger on average than winter females (t(10) = 2.989, p = 0.0136). Despite this statistical difference in size between seasons, all subjects sampled for this study outside the breeding season were well above the size range for sexually mature females (SL ≥ 8.5 cm) (see [18]). In addition, fiber density was calculated by dividing total area by size of the ROI and cell counts were standardized per section, thereby controlling for any potential measurement confounds due to variation in body size.

### Central auditory nuclei

Table 1 summarizes differences in TH-ir fiber density in nuclei throughout the central auditory system. In all cases, any significant differences in integrated intensity could be explained by differences in TH-ir fiber density and in no cases did we find differences in average intensity (intensity independent of TH-ir area) in any measured nucleus. Therefore, we present here only data that reflects differences in fiber structure (i.e. density). For each figure, micrographs from each season were selected based on representative images at comparable anatomical levels within each nucleus. Values reported in figure legends represent the mean value of that animal from measurements taken from all sections sampled throughout that particular nucleus, inclusive of the depicted image.

**Table 1:** Seasonal comparison of TH-ir fiber density in the auditory system.

Auditory Nucleus	Winter	Summer	***P***-value*
Dorsolateral division of the descending octaval nucleus + medial octavolateralis nucleus	↑	↓	0.029
Ventral secondary octaval nucleus	**-**	**-**	0.733
Octavolateralis efferent nucleus	↓	↑	0.0001
Torus semicircularis	**-**	**-**	0.297
Central posterior nucleus of the thalamus	↓	↑	0.03
Lateral division of nucleus preglomerulosus	↓	↑	0.0003
Anterior tuberal nucleus	**-**	**-**	0.201

Arrows indicate direction of significant differences between reproductive (summer) and non-reproductive (winter) females.

TH-ir, tyrosine hydroxylase immunoreactivity.

*P-values represent results from 2-tailed independent groups t-test.

**Fig 3:**
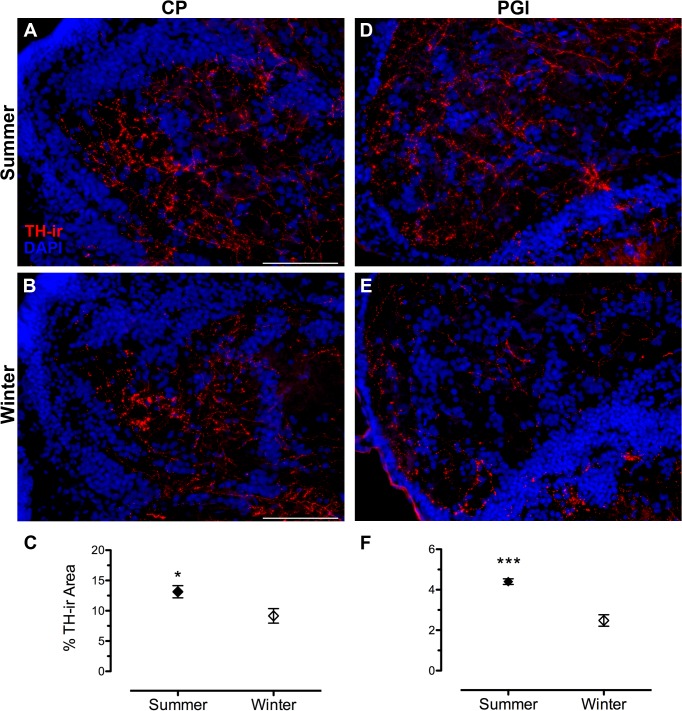
Seasonal differences in TH-ir fiber density in forebrain auditory nuclei. (A, B) central posterior nucleus of the thalamus (CP) and (D, E) lateral division of nucleus preglomerulosus (PGl). Left edge in A and B is midline of brain. Left edge in D and E is lateral edge of brain. Data in C and F are represented as percent area of the nucleus that contains TH-ir (mean ± SE). Mean TH-ir density for animal in A = 9.9%; B = 5.8%; D = 4.5%; E = 1.9%. *p = 0.03; *** *p* = 0.0003. Scale bar = 100 ¼m.

**Fig 4:**
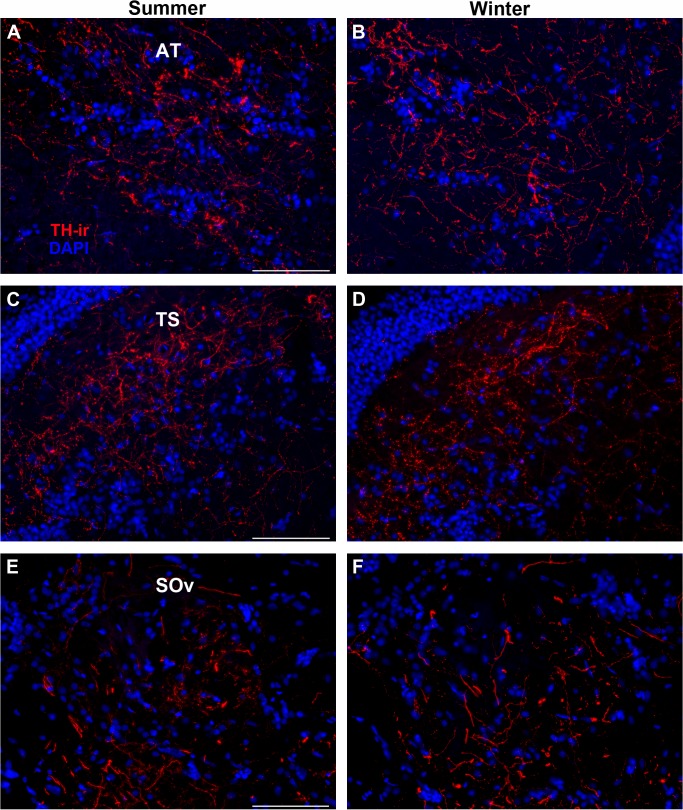
Example micrographs showing no seasonal differences in TH-ir fiber density in transverse sections through (A, B) anterior tuberal hypothalamus (AT), (C, D) torus semicircularis (TS) and (D, E) ventral division of secondary octaval nucleus (SOv). Mean ± SE TH-ir density for summer vs. winter: AT (15.22% ± 0.74/ 11.68% ± 2.7); TS (16.30% ± 1.5/ 14.28% ± 1.0); SOv (4.12% ± 0.44/ 3.94% ± 0.25). Scale bar = 100 μm.

**Fig 5:**
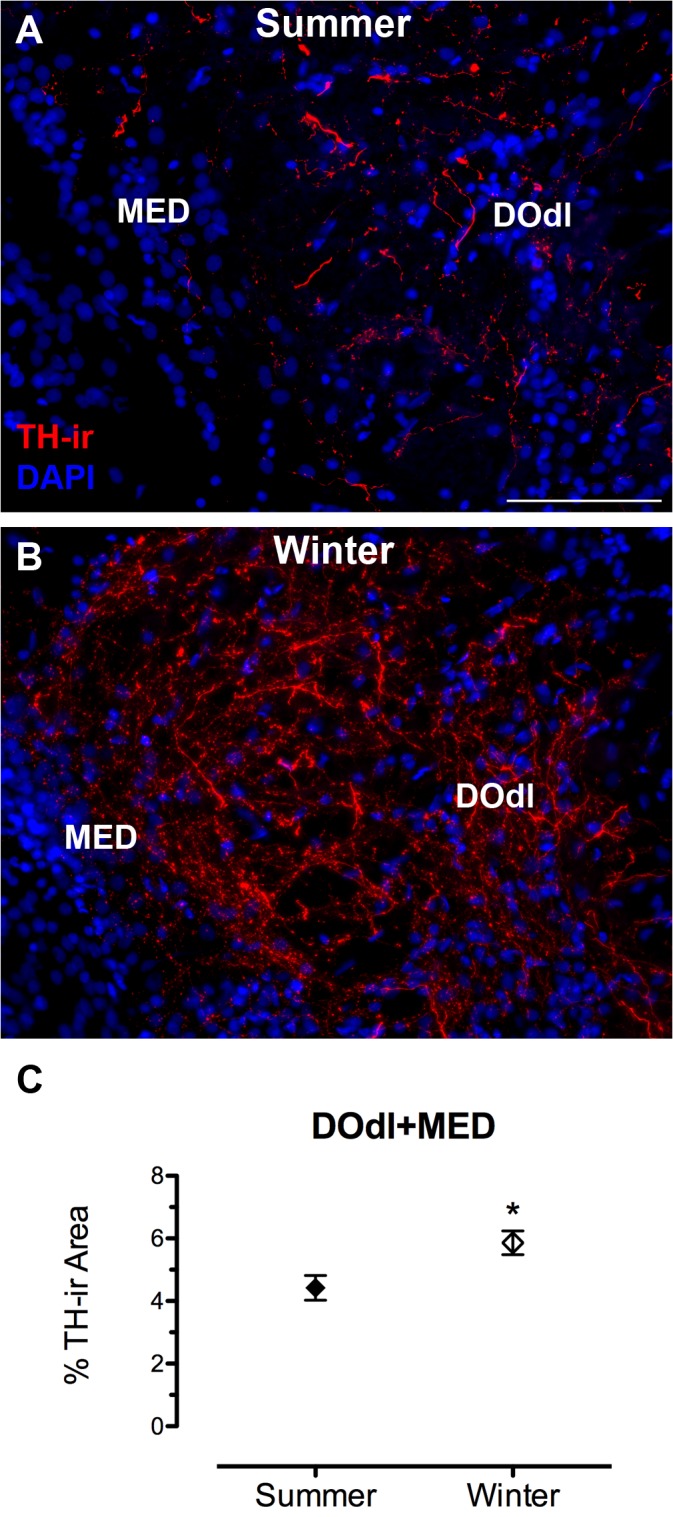
Seasonal difference in TH-ir fiber density in the auditory hindbrain. (A, B) Transverse sections through the area that includes the dorsolateral division of the descending octaval nucleus (DOdl) and medial octavolateralis nucleus (MED). The left edge in A and B is the lateral part of the fourth ventricle. Data in C are represented as percent area of the analyzed region that contains TH-ir (mean ± SE). Mean TH-ir density for animal in A = 4.3%; B = 6.6%. *p = 0.03. Scale bar = 100 μm.

To understand the overall effect of season on TH-ir density throughout the central auditory pathway, a two-way ANOVA was utilized with region of interest (DOdl+MED, SOv, OE, TS, AT, CPc, PGl) and season (winter, summer) as factors. Overall, there were significant effects of reproductive state (F_(1, 61)_ = 12.999, p = 0.001) and region of interest (F_(6, 61)_ = 76.912, p < 0.0001). There was also a significant interaction between the two main effects (F_(6, 61)_ = 4.174, p = 0.001). Summer females showed greater TH-ir fiber density in higher auditory processing areas such as the thalamic CP (t(9) = 2.566, p = 0.03) and PGl (t(8) = 6.059, p = 0.0003) (Fig 3), but not in the hypothalamic AT (t(9) = 1.280, p = 0.201). No seasonal differences in TH-ir fiber density were found in the midbrain (TS) (t(10) = 1.1, p = 0.297) or in the SOv in the hindbrain (t(8) = 0.353, p = 0.733) (Fig 4). In contrast, winter females had greater TH-ir fiber density within the primary auditory hindbrain, specifically in a region measured as DOdl+MED (t(8) = 2.647, p = 0.029) (Fig 5).

### Octavolateralis efferent nucleus

**Fig 6:**
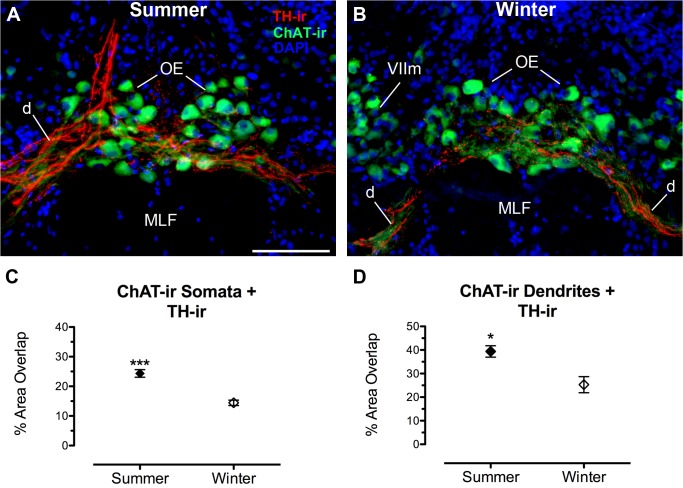
Seasonal difference in TH-ir innervation of the cholinergic octavolateralis efferent nucleus (OE). (A, B) Transverse sections through the rostral OE showing TH-ir fibers and terminals on somata and dendrites (d) of OE neurons labeled by choline acetyltransferase (ChAT)-ir. Data in C and D are expressed as the percent area of ChAT-ir in the OE that is covered by TH-ir fibers (mean ± SE). Mean percent area TH-ir overlap of OE somata and dendrites for animal in A = 22.4% and 33.4%; B = 17% and 29.7%, respectively. *p = 0.01, *** *p* = 0.0001. Abbreviations: MLF, medial longitudinal fasciculus; VIIm, facial motor nucleus. Scale bar = 100 μm.

Compared to winter females, summer females had greater TH-ir fiber density within the boundaries of the hindbrain OE nucleus (defined by the area containing ChAT-ir somata) (t(9) = 5.723, p < 0.0001). Double-labeling the OE with ChAT-ir allowed for visualization and analysis of TH-ir innervation and its putative contacts on cholinergic somata and their ventrolateral dendritic field (Fig 6). Summer females had a greater percentage of ChAT-ir somata area contacted by TH-ir fibers (t(9) = 6.471, p = 0.0001), as well as a greater percentage of ChAT-ir dendritic area contacted by TH-ir fibers (t(9) = 3.240, p = 0.01) (Fig 6).

### Saccular epithelium

**Fig 7:**
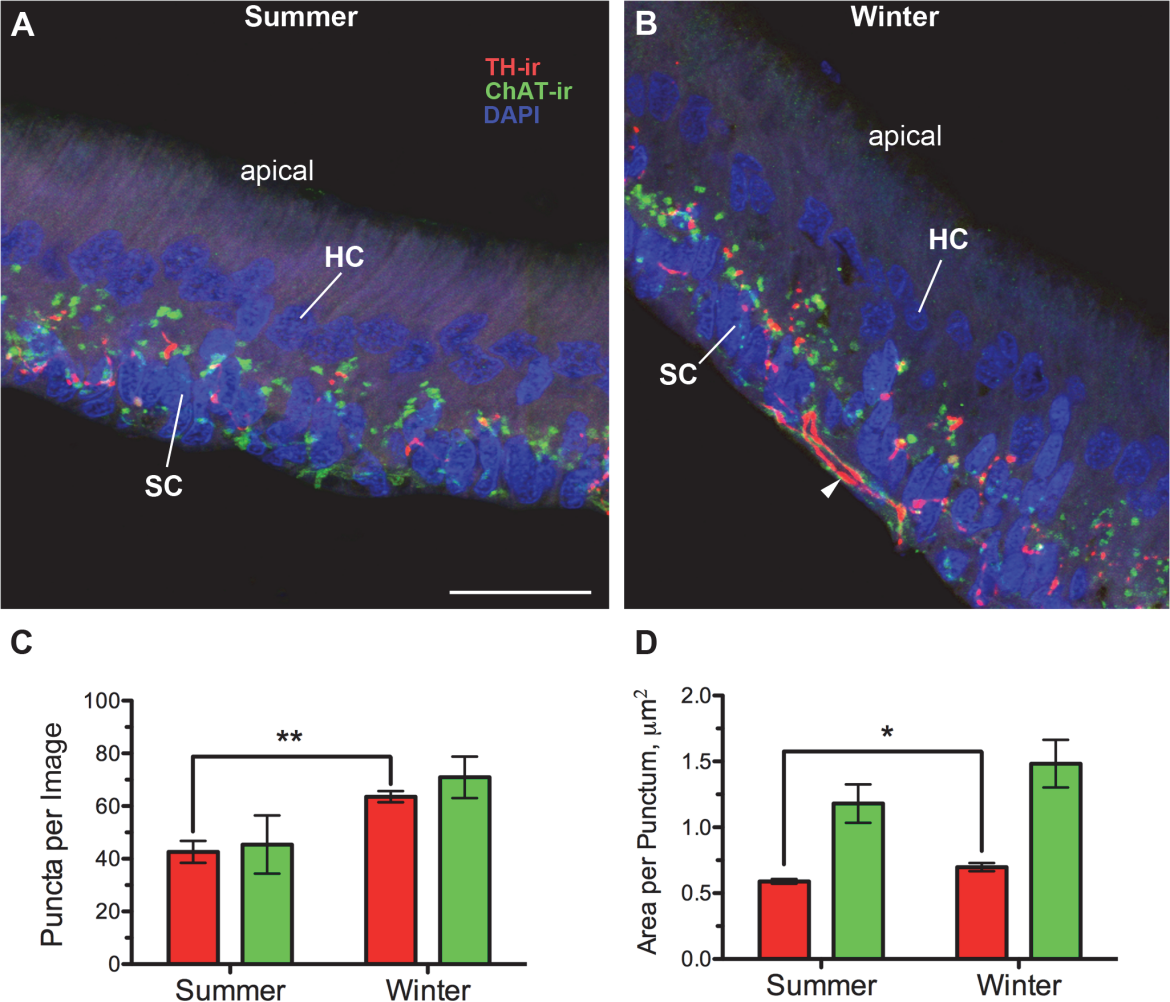
Seasonal differences in dopaminergic but not cholinergic innervation of the saccule, the main endorgan of hearing. (A, B) Transverse sections through the saccular epithelium. TH-ir and ChAT-ir puncta are largely concentrated at the base of hair cells and within the support cell layer. HC and SC labels point to DAPI-stained nuclei of individual hair cells and support cells, respectively. The rest of the hair cell is unlabeled and is a light purple background. Stereocillia (unlabeled) are located at the apical end of the hair cells. Quantification of numbers (C) and size (D) of putative TH-ir and ChAT-ir terminals (puncta) in the saccule (mean ± SE). Arrowhead in B indicates an example of a thick, varicose TH-ir fiber along the base of the SC layer that was excluded from the puncta analysis. Colors in the graphs match TH-ir and ChAT-ir in the micrographs. Mean number of puncta per image for animal in A = 41.4; B = 68.7. Mean area per punctum for animal A = 0.63 μm^2^; B = 0.78 μm^2^. *p = 0.017, **p = 0.001. Scale bar = 25 μm.

Cholinergic efferent innervation from OE (hindbrain) and dopaminergic innervation from diencephalic TPp can be seen at the base of hair cells in the saccule (Fig 7; also see [24]). In order to focus on putative terminals from both sets of efferents, we quantified TH-ir and ChAT-ir puncta and excluded thick—ir fibers characteristic of efferent axons entering the base of the saccular epithelium (Fig 7B). Winter, non-reproductive females had a greater number of TH-ir puncta per section (t(9) = 4.4710, p = 0.001), as well as a greater area per TH-ir punctum (t(9) = 2.933, p = 0.017) (Fig 7). In contrast, no differences were found between seasons with regard to ChAT-ir puncta in the SE (Table 2).

**Table 2:** Seasonal comparison of dopaminergic and cholinergic efferent fiber terminals in the saccule (main endorgan of hearing).

Measurement	Winter	Summer	***P***-value*
Puncta per image			
TH-ir	↑	↓	0.001
ChAT-ir	**-**	**-**	0.086
Area per punctum			
TH-ir	↑	↓	0.017
ChAT-ir	**-**	**-**	0.238

Arrows indicate direction of significant differences between reproductive (summer) and non-reproductive (winter) females.

TH, tyrosine hydroxylase; ChAT, choline acetyltransferase immunoreactivity.

*P-values represent results from 2-tailed independent groups t-test.

### TH-ir cell counts

**Fig 8:**
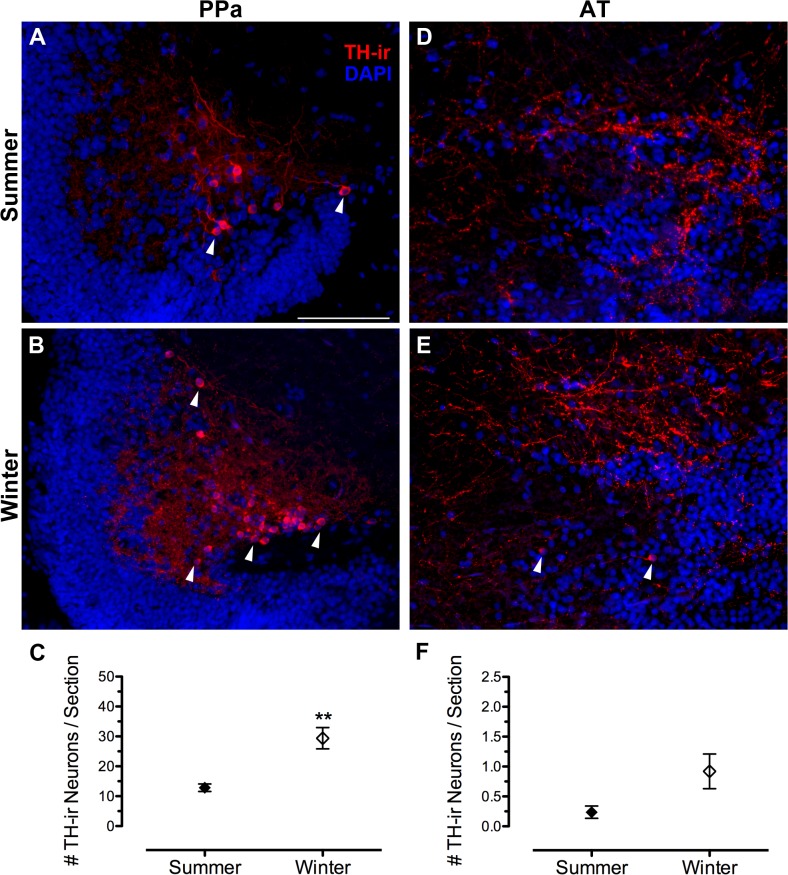
Seasonal differences in dopaminergic neuron number in the forebrain. (A, B) Clusters of small TH-ir cells (arrowheads) located in the lateral region of the anterior parvocellular preoptic nucleus (PPa). (D, E) Sparse numbers of TH-ir cells (arrowheads) in the anterior tuberal hypothalamus (AT). Data in C and D are expressed as number of TH-ir neurons per section (mean ± SE). Mean number of neurons per section for animal in A = 14; B = 32; D = 0; E = 0.6. **p = 0.001. Scale bar = 100 μm.

**Fig 9:**
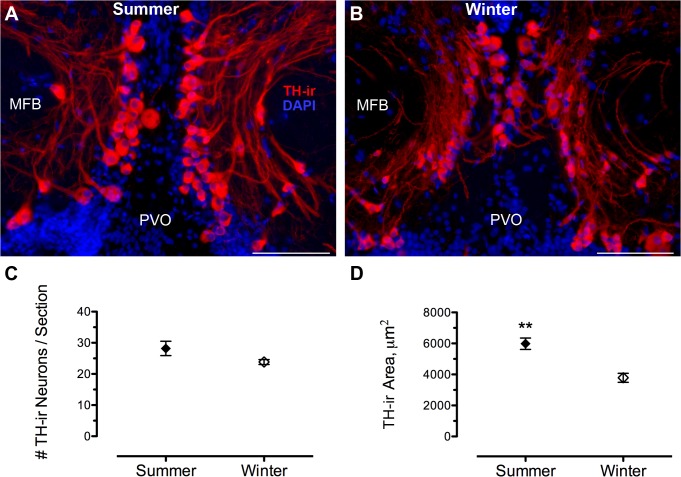
Seasonal differences in TH-ir area but not cell number in the dopaminergic periventricular posterior tuberculum (TPp). (A, B) Large, pear-shaped TH-ir neurons and thick processes are seen just dorsal and lateral to the paraventricular organ (PVO) and medial to the medial forebrain bundle (MFB) along the midline in transverse section through the diencephalon. Data in C are expressed as number of TH-ir neurons per section (mean ± SE). (D) TH-ir area including cells and processes are quantified per unit area (143,139 μm^2^). Mean TH-ir area for animal in A = 6205.1μm^2^; B = 2658.4 μm^2^. **p = 0.001. Scale bar = 100 μm.

**Fig 10:**
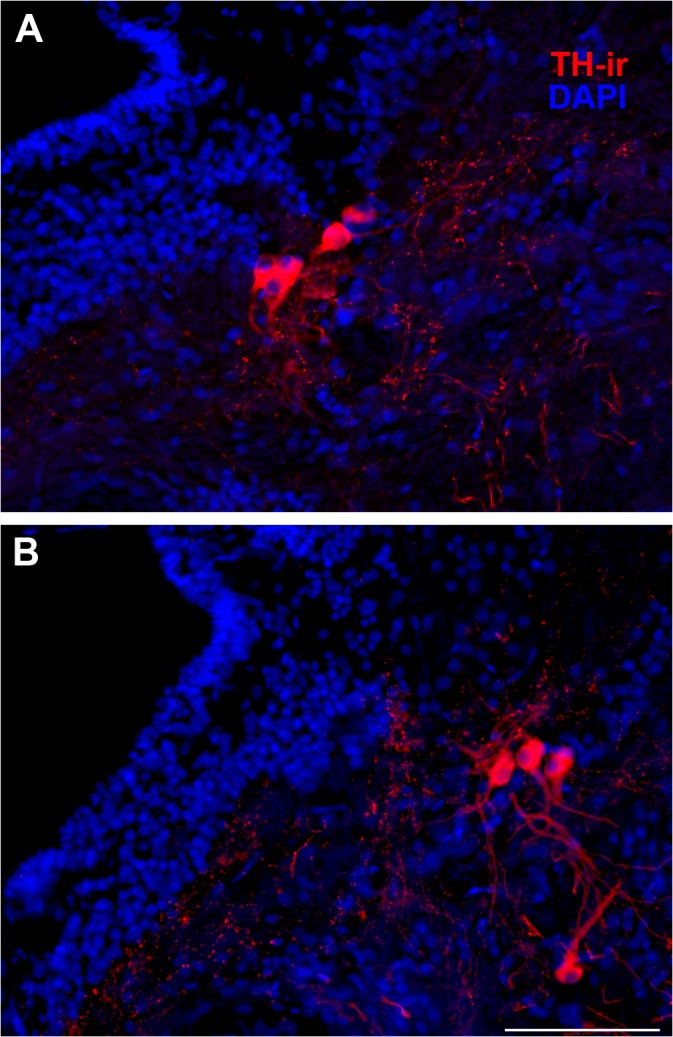
No seasonal differences in number of TH-ir cells in the noradrenergic locus coeruleus (LC). Examples of small numbers of clustered TH-ir neurons just lateral to the fourth ventricle (IV) characteristic of the LC in transverse sections through the isthmal brainstem in summer (A) and winter (B) animals. Mean ± SE for number of TH-ir cells/ section = 4.2 ± 0.37 (summer) and 4.7 ± 0.21 (winter). Scale bar = 100 μm.

Table 3 summarizes seasonal comparisons of numbers of TH-ir neurons in PPa, AT, TPp and LC between reproductive and non-reproductive female midshipman. Within the PPa, non-reproductive, winter females had twice the average number of TH-ir (dopaminergic) neurons per section (29.4 ± 3.5) compared to summer gravid females (12.8 ± 1.3) (t(9) = 4.738, p = 0.0011) (Fig 8A–8C). Similarly, although AT had relatively few TH-ir neurons per section, winter females had greater numbers (0.92 ± 0.29) compared to summer females (0.24 ± 0.10) (t(9) = 2.395, p = 0.04) (Fig 8D–8F), but the significance of this finding is questionable given that there was an unequal number of sections sampled between groups in this small area (see Methods). The number of TH-ir neurons within the dopaminergic TPp was not statistically different in summer (28.2 ± 2.3) vs. winter (23.8 ± 0.80) (t(9) = 1.930, p = 0.086). However, when the total TH-ir area within the TPp was quantified which included the large pear-shaped neurons and their proximal thick processes, summer females had a greater TH-ir area (5981.27 ± 366.60 μm^2^) compared to winter females (3784.52 ± 292.53 μm^2^) (t(10) = 4.684, p = 0.001) (Fig 9). Finally, no differences were found in the number of TH-ir neurons within the LC between seasons (t(9) = 1.137, p = 0.2848) (Fig 10).

**Table 3:** Seasonal comparison of catecholaminergic neuron number.

TH-ir cell group	Winter	Summer	***P***-value*
Locus coeruleus	**-**	**-**	0.284
Periventricular posterior tuberculum	**-**	**-**	0.086
Anterior tuberal hypothalamus	**-**	**-**	0.040^†^
Anterior parvocellular preoptic nucleus	↑	↓	0.0011

Arrows indicate direction of significant differences between reproductive (summer) and non-reproductive (winter) females.

TH, tyrosine hydroxylase.

*P-values represent results from 2-tailed independent groups t-test.

^†^Statistical difference is uncertain due to an unequal number of sections sampled between groups.

## Discussion

Here we report seasonal differences in CA fiber innervation of several areas in the midshipman auditory pathway that vary with reproductive state. To our knowledge this is the first study to report natural seasonal plasticity of TH-ir innervation in the central and peripheral auditory system of a vertebrate. These findings support a role for catecholamines as important modulators of auditory plasticity and behavior that varies with reproductive state in midshipman. Interestingly, seasonal effects were bidirectional: specific forebrain auditory areas show greater TH-ir fiber innervation in the summer reproductive season while primary auditory hindbrain and inner ear show greater TH-ir fiber density in the winter non-reproductive season. Hypotheses for functional significance of these differences will be discussed below.

Because summer females were taken from nests and sacrificed that evening, they were likely exposed to male mate calls within 24 hours of sacrifice. However, studies in songbirds indicate that exposure to song does not affect TH-ir, only pTH-ir, the phosphorylated, or “active” form, of TH [45]. Therefore, although CA neurons are activated by hearing song [40, 45, 46], TH-ir itself appears stable under acute conditions and seasonal differences are most likely indicative of longer term physiological transitions to opposing reproductive states.

While biochemical assays show a positive relationship between TH activity and reproductive cycle in major brain regions (telencephalon, hypothalamus, medulla) in female catfish [47], few studies have investigated neuroanatomical changes in CA neurons or their projections outside the hypothalamus in relation to reproductive state. Heimovics et al. [48] found that male song sparrows in breeding condition have greater pTH-ir in lateral septum compared to non-breeding males, but pTH-ir was not found in the auditory forebrain (i.e., caudomedial nidopallium) and was not investigated in other auditory nuclei. Hypothalamic dopaminergic A12/A14 neurons which are known to control the pituitary gonadal axis across vertebrates [35, 49] are predicted to be dynamic in species with seasonal reproduction. The equivalent DA neurons in teleosts lie in the PPa and these neurons are known to directly project to the anterior pituitary to inhibit gonadotropin release in several species [43, 50, 51]. Thus, our results that show winter non-reproductive females have more than double the number of TH-ir PPa neurons compared to summer reproductive females are consistent with the role of this specific population of dopaminergic neurons in other teleosts as inhibitory for the reproductive axis. In trout, these DA neurons in the PPa all express ERα [52] and midshipman, like other teleosts, have abundant ERα and ERβ1/2 in this area [53, 54] as well as androgen receptors [55], making these particular DA neurons potentially direct targets of seasonal increases in estrogen and/or testosterone that occur prior to spawning in females [18]. Estrogens and androgens are documented to have differential and species-specific effects on TH mRNA expression in teleost fishes [56, 57].

### Possible mechanisms underlying seasonal differences in TH-ir fiber density

The present findings suggest that seasonal changes in circulating steroid hormone levels [18] may directly target and regulate CA synthesis in specific neuronal populations. In support of this, the TPp is a steroid-sensitive brain region as it is replete with both androgen and estrogen receptors [53–55]. In addition, this area contains abundant aromatase mRNA and protein: a site where conversion of androgen to estrogen occurs [54, 58]. A previous tract tracing study [24] confirmed that a subset of TH-ir TPp neurons project directly to the saccule and strong immunohistochemical evidence suggests that neurons from this same area project to OE and CP. Whether the same DAergic neurons innervate multiple auditory nuclei is unknown, but descending projections that branch off to exit nerve VIII en route to the saccule appear to continue caudally and heavily innervate OE (see [24]). However, only about 5–10% of TH-ir neurons in TPp send descending projections to the saccule [24]. Therefore it is likely that nearby diencephalic auditory areas such as PGl and CP receive ascending or local projections from a separate group of TH-ir neurons in TPp. Ultimately, double-label experiments will need to confirm steroid receptor expression and type in TH-ir neurons and if those TH-ir populations project to auditory nuclei. Although we did not find a significant difference in number of TH-ir TPp neurons between seasons, the total TH-ir area in the TPp including proximal neurites and somata was greater in summer females and supports these cells as targets of steroid or seasonal-mediated plasticity.

Similarly, CA neurons in the canary ventral tegmental area (VTA) and LC express ERα [59] and these neurons as well as the substantia nigra likely contribute CA innervation to auditory nuclei (see [4]). Other studies in songbirds have experimentally manipulated circulating steroid hormones to mimic changes in reproductive state and have demonstrated steroid regulation of TH-ir fiber density in forebrain and midbrain auditory nuclei [7, 8, 45, 60]. However, unlike songbirds, circulating E2 levels are no different in gravid midshipman females sampled from nest sites compared to non-reproductive females. Rather, E2 as well as T levels peak in the pre-spawning period during gonadal recrudescence [18]. Therefore, while pre-spawning elevation in circulating steroid hormones may serve to initially alter gene expression of TH in neurons that project to auditory nuclei, the differences in TH-ir we found cannot be attributed to blood-borne steroids at time of sacrifice, but likely to other physiological factors that underlie reproductive readiness ([9, 10]; see below). However, brain aromatase mRNA, which is regulated by circulating steroids and fluctuates seasonally, is upregulated during the pre-spawning period and may serve to maintain local brain levels of estrogen during the summer spawning period when gonadal sources of E2 are low [10, 18, 61–63]; this local estrogen production may be maintained by positive feedback [64], and in turn increase or decrease TH-ir (see below).

In support of a decoupling between circulating hormone levels and CA fiber densities, Pawlisch et al. [65] simulated spring (breeding) and fall (non-breeding) conditions in European starlings by subjecting females to long day lengths with E2 implants and periodic male courtship song or short day length with no supplemental E2 and fall (non-courtship) song, respectively. Interestingly, they found spring-conditioned females that occupied nests had higher TH-ir density in the nucleus accumbens (forebrain DA target for reward-related behavior) and higher density of dopamine-beta hydroxylase-ir (DBH, rate-limiting enzyme for noradrenaline synthesis) in the ventromedial hypothalamus compared to spring conditioned females that did not occupy nests. Since E2 levels in spring-conditioned females were equivalent regardless of nest occupancy, these results suggest differences in CA fiber density were due to reproductive readiness, not hormone levels [65]. Likewise, in the present study, all reproductive summer females were highly gravid and collected from nests in the wild and thus were sampled in a state of reproductive readiness. As stated above, these females were previously shown to have equivalent levels of circulating E2 as winter, non-reproductive females [18].

Direct or indirect environmental influence on TH synthesis (and thus TH-ir density) may occur through melatonin signaling. Both light and temperature are known to influence melatonin release from fish pineal gland [66] and melatonin regulation of TH gene expression and activity has been demonstrated in teleosts [67, 68]. Although melatonin receptors have not been colocalized to CA neurons in teleosts, 2-[^125^I]-melatonin binding sites are high in the diencephalon and telencephalon where most DA neurons are located [68, 69]. In birds and mammals there is neuroanatomical evidence for direct melatonin action on dopaminergic neurons. In the avian hypothalamus, dopamine neurons which also produce melatonin and contain melanopsin, a circadian photoreceptor, are the direct conduits for synchronizing environmental light cycle and regulation of reproductive cycle via regulation of gonadotropin-releasing hormone [70, 71]. Furthermore, dopaminergic neurons in the VTA in rodents contain melatonin receptors [72] providing neuroanatomical evidence for a link between seasonal changes in light cycle and DA function.

### Differences in TH-ir density vary by area and season

We demonstrate that specific forebrain auditory areas (CP, PGl) and the OE show greater TH-ir fiber innervation in the summer reproductive season while the primary auditory hindbrain (DOdl+MED) and saccular epithelium show greater TH-ir fiber density in the winter non-reproductive season. Other regions in hindbrain (SOv), midbrain (TS) and hypothalamus (AT) showed no difference in TH-ir density between seasons. Differential effects of reproductive state on TH-ir density by region may result from regulation of separate TH-ir neuronal populations via different hormones or receptors (see [57, 73]). These CA neurons that are seasonally plastic may be different neurons within a single nucleus (e.g., TPp, above), or neurons from DA vs NA cell populations that contribute TH-ir projections to the same nuclei. For instance, while E2 appears to upregulate TH-ir fiber density in auditory nuclei in white-throated sparrows [7, 8], non-breeding male white-throated sparrows treated with testosterone show a decrease in NA (DBH-ir) fiber innervation in the auditory midbrain [60]. Similarly, seasonal studies in songbirds have investigated densities of α_2_-noradrenergic receptors in song control nuclei, lateral septum and medial preoptic area and found inverse relationships to T levels, song control volume, breeding condition and singing behavior [74, 75]. Since we did not measure DBH-ir in the current study, we cannot resolve the contribution of the NA system to the TH-ir fiber densities that were measured. It is noteworthy that we did not find a seasonal difference in number of LC neurons or in fiber density of the TS, one of its known efferent targets [42].

### Functional implications of seasonal plasticity of TH-ir innervation

#### Peripheral auditory system

As mentioned previously, seasonal and hormone-induced peripheral auditory plasticity is well documented in both female and male midshipman [14–17]. Interestingly, Sisneros and Bass [16] first discovered that along with the ability of primary afferent neurons to encode higher frequencies, summer females also showed a dramatic increase in auditory sensitivity (gain). The recent study by Rohmann et al. [20] demonstrates that seasonal change in number of BK channels in saccular hair cells can ultimately function to allow for encoding of higher frequencies as well as increasing auditory sensitivity (i.e., ability to encode at lower stimulus thresholds). However, it has also been suggested that seasonal changes in reproductive state may modulate the octavolateralis efferent system which in turn may also affect saccular hair cell sensitivity [10, 15, 16]. The current study now provides neuroanatomical evidence that dopaminergic TPp neurons may seasonally modulate the sensitivity of the inner ear directly at the level of the hair cell, or indirectly by modulating the cholinergic OE. Prior to our recent study, TH-ir terminals had not been reported in association with non-mammalian vertebrate auditory hair cells [24]. Therefore, interpretations on DA action on hair cell or primary afferent physiology can only be speculated based on mammalian data. In mammals, DA neurons and their projections make up part of the lateral olivocochlear (LOC) efferent system that synapses onto primary afferent dendrites of inner hair cells [76–79]. Studies in rodents (mainly guinea pigs) have demonstrated that dopamine has an inhibitory effect on auditory nerve physiology, namely a decrease in sound-evoked compound action potentials and an increase in single unit thresholds without affecting hair cell function or frequency tuning [80, 81]. However, several pharmacological studies have revealed paradoxical physiological effects of both D1 and D2 receptor subtypes in the cochlea which may be dependent on complex pre- and postsynaptic interactions between the efferent and afferent processes [79, 80, 82–85]. If, as in mammals, dopamine is largely inhibitory at the level of the primary afferent dendrites in midshipman, then a decrease in number of TH-ir terminals in the summer reproductive season may indicate less DA inhibition and thus increase overall sensitivity of the saccule, consistent with seasonal physiological data recorded from primary afferents [16].

An additional layer of DA modulation of the saccule may be indirect via the cholinergic OE which is documented to have an inhibitory effect on fish saccular auditory hair cells [86]. In support of an inhibitory function, OE somata were found to be γ-Aminobutyric acid (GABA)-ir in the toadfish, *Opsanus tau*, a close relative of midshipman (family, Batrachoididae) [87]. This pattern is consistent with extensive colocalization of cholinergic and GABAergic markers of LOC efferents in mammals [88]. Interestingly, OE neurons appear to inhibit hair cells but excite primary afferents in the vestibular system of toadfish [89]. If CA innervation is inhibitory on OE, this would result in release of cholinergic inhibition in the saccule in the summer, and again, increased sensitivity in the reproductive season. However, Filippi et al. [90] report that equivalent DA neurons in TPp of zebrafish co-express vesicular glutamate transporter (vGlut) and are therefore glutamatergic. If indeed DA projections from the TPp are excitatory and comprise the majority of TH-ir input onto inhibitory cholinergic OE neurons, then increased TH-ir innervation onto OE in the summer may result in the potential for increased inhibition of the saccule. However, multiple D1 and D2 receptor subtypes have been identified in hair cell preparations from trout saccule [91] as well as in the OE of European eel [92, 93], and, as is the case in mammals, multiple DA receptors make for more complex modulation scenarios (e.g., [79]). Ultrastructural and/or receptor subtype localization to pre or postsynaptic structures would give anatomical insight into DA function at both the OE and sensory receptor level. For instance, seasonal differences in expression of excitatory (vs. inhibitory) DA receptor-linked signaling pathways might have direct effects on saccular primary afferents.

Prior studies have utilized acetylcholinesterase histochemistry to identify cholinergic efferents on saccular hair cells in other teleosts [94, 95]. The present study confirms localization of cholinergic terminals at the base of saccular hair cells by ChAT-ir and our double-label findings are consistent with the mammalian condition that cholinergic and dopaminergic neurons comprise separate efferent populations [77, 96]. In addition, efferent synapses have been identified on goldfish saccular afferents and vesicles in those terminals are similar to cholinergic synapses on the base of hair cells [97]. Therefore, DA may function, in part, to modulate acetylcholine release not only from ChAT-ir terminals at the base of hair cells, but also at efferent-afferent synapses. This cholinergic efferent action may serve to increase signal to noise [98, 99] for detection of conspecific acoustic signals in the seasonally changing soundscape. For instance, the rocky intertidal zone where midshipman nests are found is dense with type I male courtship at night [100], therefore, selective inhibition of hair cells encoding frequencies outside the spectrum of the advertisement call may provide additional filtering to detect relevant social signals.

#### Central auditory system

Here we demonstrate that TH-ir innervation in forebrain (CP and PGl) and hindbrain (DOdl+MED) auditory areas vary differentially with reproductive state and may provide a modulatory substrate for seasonal changes in central auditory response properties which is unexplored at this time in midshipman. To date, a single study has directly investigated the response properties of the auditory thalamus (CP) in fish and suggests its function is to encode broad spectrum complex natural stimuli, as single units do not phase lock to tones like lower processing areas [101]. While the fundamental frequency of the midshipman advertisement call is tonal, field recordings show multiple harmonics of equivalent intensity from individual hums which are thought to confer important sound source localization and potentially mate quality information [11, 16, 102]. Thus, the CP is likely involved in discrimination of conspecific vocalizations. In support of this, Petersen et al. [40] recently demonstrated a strong cFos-ir response in the CP of type I males exposed to conspecific advertisement calls compared to ambient noise. In addition, they found a significant positive correlation between numbers of cFos-ir neurons in CP and percent colocalization of cFos-ir in TH-ir TPp neurons, which further supports CP as a dopaminergic target of TPp [24]. Although even less is known about its physiology, PGl, like CP, receives direct projections from TS [25] and is thought to provide the major ascending auditory information to the dorsal medial telencephalon (Dm) [29], while CP has strong reciprocal connections to both Dm and ventral telencephalic areas [27].

We recently demonstrated putative TH-ir contacts on auditory neurons in multiple hindbrain nuclei defined by neurobiotin labeling of the saccule [24]. However, many of those nuclei are comprised of small clusters of cells not easily defined by general counterstain and were less amenable for analysis in the present study [25, 26]. The dorsal hindbrain contains octaval sensory nuclei in well delineated areas and contains dense TH-ir innervation in midshipman (Fig 2H; [24, 103]). The hindbrain region sampled that includes DOdl and MED showed greater TH-ir density in the non-reproductive period compared to the reproductive summer. Interestingly, this pattern is similar to the saccule, but opposite to that found in forebrain areas (CP and PGl). DOdl is an octaval integration site as it receives direct afferent input from the saccule as well as lateral line input and is interconnected with MED, the major lateral line recipient in the hindbrain [25, 44]. Importantly, primary afferents of the posterior lateral line system of midshipman are able to encode frequencies that overlap with the male advertisement call [104].

## Conclusions

Seasonal changes in CA innervation of the peripheral and central auditory system in midshipman females may contribute to auditory plasticity and behavior that varies with reproductive state. The functional significance of opposing plasticity in CA input at primary vs. higher auditory processing centers is unclear, but may be related to differentially modulating gain to 1) optimize signal detection in a noisy environment, characteristic of the summer nesting grounds in the rocky intertidal, 2) increase discrimination, perception and salience of species-specific vocalizations (e.g., [105, 106]; see [4, 5]) and 3) coordinate auditory responsiveness with the motivated motor response and decision to localize a potential mate’s nest.

## References

[1] Berridge CW, Waterhouse BD. The locus coeruleus-noradrenergic system: modulation of behavioral state and state-dependent cognitive processes. Brain Res Brain Res Rev. 2003;42: 33–84. 12668290 10.1016/s0165-0173(03)00143-7

[2] Hurley LM, Devilbiss DM, Waterhouse BD. A matter of focus: monoaminergic modulation of stimulus coding in mammalian sensory networks. Curr Opin Neurobiol. 2004;14: 488–495. 15321070 10.1016/j.conb.2004.06.007

[3] Joshua M, Adler A, Bergman H. The dynamics of dopamine in control of motor behavior. Curr Opin Neurobiol. 2009;19: 615–620. 10.1016/j.conb.2009.10.001 19896833

[4] Maney DL. The incentive salience of courtship vocalizations: hormone-mediated 'wanting' in the auditory system. Hear Res. 2013;305: 19–30. 10.1016/j.heares.2013.04.011 23665125

[5] Caras ML. Estrogenic modulation of auditory processing: a vertebrate comparison. Front Neuroendocrinol. 2013;34: 285–299. 10.1016/j.yfrne.2013.07.006 23911849 PMC3788044

[6] Maney DL, Pinaud R. Estradiol-dependent modulation of auditory processing and selectivity in songbirds. Front Neuroendocrinol. 2011;32: 287–302. 10.1016/j.yfrne.2010.12.002 21146556 PMC3119742

[7] LeBlanc MM, Goode CT, MacDougall-Shackleton EA, Maney DL. Estradiol modulates brainstem catecholaminergic cell groups and projections to the auditory forebrain in a female songbird. Brain Res. 2007;1171: 93–103. 17764666 10.1016/j.brainres.2007.06.086

[8] Matragrano LL, Sanford SE, Salvante KG, Sockman KW, Maney DL. Estradiol-dependent catecholaminergic innervation of auditory areas in a seasonally breeding songbird. Eur J Neurosci. 2011;34: 416–425. 10.1111/j.1460-9568.2011.07751.x 21714815 PMC3148281

[9] Forlano PM, Bass AH. Neural and hormonal mechanisms of reproductive-related arousal in fishes. Horm Behav. 2011;59: 616–629. 10.1016/j.yhbeh.2010.10.006 20950618 PMC3033489

[10] Forlano PM, Sisneros JA, Rohmann KN, Bass AH. Neuroendocrine control of seasonal plasticity in the auditory and vocal systems of fish. Front Neuroendocrinol. 2014;in press. 10.1016/j.yfrne.2014.08.002 PMC4342331 25168757

[11] Sisneros JA. Steroid-dependent auditory plasticity for the enhancement of acoustic communication: recent insights from a vocal teleost fish. Hear Res. 2009;252: 9–14. 10.1016/j.heares.2008.12.007 19168118 PMC2698033

[12] Bass AH. Shaping brain sexuality. American Scientist. 1996;84: 352–363.

[13] Bass AH, McKibben JR. Neural mechanisms and behaviors for acoustic communication in teleost fish. Prog Neurobiol. 2003;69: 1–26. 12637170 10.1016/s0301-0082(03)00004-2

[14] Rohmann KN, Bass AH. Seasonal plasticity of auditory hair cell frequency sensitivity correlates with plasma steroid levels in vocal fish. J Exp Biol. 2011;214: 1931–1942. 10.1242/jeb.054114 21562181 PMC3092729

[15] Sisneros JA. Seasonal plasticity of auditory saccular sensitivity in the vocal plainfin midshipman fish, *Porichthys notatus* . J Neurophysiol. 2009;102: 1121–1131. 10.1152/jn.00236.2009 19553489

[16] Sisneros JA, Bass AH. Seasonal plasticity of peripheral auditory frequency sensitivity. J Neurosci. 2003;23: 1049–1058. 12574435 10.1523/JNEUROSCI.23-03-01049.2003 PMC6741921

[17] Sisneros JA, Forlano PM, Deitcher DL, Bass AH. Steroid-dependent auditory plasticity leads to adaptive coupling of sender and receiver. Science. 2004;305: 404–407. 15256672 10.1126/science.1097218

[18] Sisneros JA, Forlano PM, Knapp R, Bass AH. Seasonal variation of steroid hormone levels in an intertidal-nesting fish, the vocal plainfin midshipman. Gen Comp Endocrinol. 2004;136: 101–116. 14980801 10.1016/j.ygcen.2003.12.007

[19] Coffin AB, Mohr RA, Sisneros JA. Saccular-specific hair cell addition correlates with reproductive state-dependent changes in the auditory saccular sensitivity of a vocal fish. J Neurosci. 2012;32: 1366–1376. 10.1523/JNEUROSCI.4928-11.2012 22279221 PMC3564634

[20] Rohmann KN, Fergus DJ, Bass AH. Plasticity in ion channel expression underlies variation in hearing during reproductive cycles. Curr Biol. 2013;23: 678–683. 10.1016/j.cub.2013.03.014 23562266 PMC3676172

[21] McKibben JR, Bass AH. Behavioral assessment of acoustic parameters relevant to signal recognition and preference in a vocal fish. J Acoustic Soc Am. 1998;104: 3520–3533. 9857511 10.1121/1.423938

[22] Zeddies DG, Fay RR, Alderks PW, Shaub KS, Sisneros JA. Sound source localization by the plainfin midshipman fish, Porichthys notatus. J Acoust Soc Am. 2010;127: 3104–3113. 10.1121/1.3365261 21117759

[23] Remage-Healey L. Frank Beach Award Winner: Steroids as neuromodulators of brain circuits and behavior. Horm Behav. 2014;66: 552–560. 10.1016/j.yhbeh.2014.07.014 25110187 PMC4180446

[24] Forlano PM, Kim SD, Krzyminska ZM, Sisneros JA. Catecholaminergic connectivity to the inner ear, central auditory, and vocal motor circuitry in the plainfin midshipman fish *Porichthys notatus* . J Comp Neurol. 2014;522: 2887–2927. 10.1002/cne.23596 24715479 PMC4107124

[25] Bass AH, Bodnar DA, Marchaterre MA. Midbrain acoustic circuitry in a vocalizing fish. J Comp Neurol. 2000;419: 505–531. 10742718 10.1002/(sici)1096-9861(20000417)419:4<505::aid-cne7>3.0.co;2-3

[26] Bass AH, Marchaterre MA, Baker R. Vocal-acoustic pathways in a teleost fish. J Neurosci. 1994;14: 4025–4039. 8027760 10.1523/JNEUROSCI.14-07-04025.1994 PMC6577059

[27] Goodson JL, Bass AH. Vocal-acoustic circuitry and descending vocal pathways in teleost fish: Convergence with terrestrial vertebrates reveals conserved traits. J Comp Neurol. 2002;448: 298–322. 12115710 10.1002/cne.10258

[28] McCormick CA. Anatomy of the central auditory pathways of fish and amphibians In: Popper A, Fay RR, editors. Comparative hearing: fish and amphibians. New York: Springer; 1999 pp. 155–217.

[29] McCormick CA. Auditory/lateral line CNS: Anatomy In: Farrell AP, editor. Encyclopedia of Fish Physiology: From Genome to Environment. San Diego: Academic Press; 2011 pp. 283–291.

[30] Chagnaud BP, Baker R, Bass AH. Vocalization frequency and duration are coded in separate hindbrain nuclei. Nat Commun. 2011;2: 346 10.1038/ncomms1349 21673667 PMC3166519

[31] Chagnaud BP, Bass AH. Vocal corollary discharge communicates call duration to vertebrate auditory system. J Neurosci. 2013;33: 18775–18780. 10.1523/JNEUROSCI.3140-13.2013 24285884 PMC3841447

[32] Weeg MS, Land BR, Bass AH. Vocal pathways modulate efferent neurons to the inner ear and lateral line. J Neurosci. 2005;25: 5967–5974. 15976085 10.1523/JNEUROSCI.0019-05.2005 PMC6724790

[33] Schweitzer J, Lohr H, Filippi A, Driever W. Dopaminergic and noradrenergic circuit development in zebrafish. Dev Neurobiol. 2012;72: 256–268. 10.1002/dneu.20911 21567980

[34] Tay TL, Ronneberger O, Ryu S, Nitschke R, Driever W. Comprehensive catecholaminergic projectome analysis reveals single-neuron integration of zebrafish ascending and descending dopaminergic systems. Nat Commun. 2011;2: 171 21266970 10.1038/ncomms1171 PMC3105308

[35] Yamamoto K, Vernier P. The evolution of dopamine systems in chordates. Front Neuroanat. 2011;5: 21 10.3389/fnana.2011.00021 21483723 PMC3070214

[36] O'Connell LA, Hofmann HA. The vertebrate mesolimbic reward system and social behavior network: a comparative synthesis. J Comp Neurol. 2011;519: 3599–3639. 10.1002/cne.22735 21800319

[37] McLean DL, Fetcho JR. Ontogeny and innervation patterns of dopaminergic, noradrenergic, and serotonergic neurons in larval zebrafish. J Comp Neurol. 2004;480: 38–56. 15515022 10.1002/cne.20280

[38] Kittelberger JM, Bass AH. Vocal-motor and auditory connectivity of the midbrain periaqueductal gray in a teleost fish. J Comp Neurol. 2013;521: 791–812. 10.1002/cne.23202 22826153 PMC3504190

[39] Rink E, Wullimann MF. The teleostean (zebrafish) dopaminergic system ascending to the subpallium (striatum) is located in the basal diencephalon (posterior tuberculum). Brain Res. 2001;889: 316–330. 11166725 10.1016/s0006-8993(00)03174-7

[40] Petersen CL, Timothy M, Kim DS, Bhandiwad AA, Mohr RA, Sisneros JA, et al Exposure to advertisement calls of reproductive competitors activates vocal-acoustic and catecholaminergic neurons in the plainfin midshipman fish, *Porichthys notatus* . PLoS One. 2013;8: e70474 10.1371/journal.pone.0070474 23936438 PMC3735598

[41] Brantley RK, Bass AH. Cholinergic neurons in the brain of a teleost fish (*Porichthys notatus*) located with a monoclonal antibody to choline acetyltransferase. J Comp Neurol. 1988;275: 87–105. 3170792 10.1002/cne.902750108

[42] Ma PM. Catecholaminergic systems in the zebrafish. II. Projection pathways and pattern of termination of the locus coeruleus. J Comp Neurol. 1994;344: 256–269. 8077460 10.1002/cne.903440207

[43] Zohar Y, Munoz-Cueto JA, Elizur A, Kah O. Neuroendocrinology of reproduction in teleost fish. Gen Comp Endocrinol. 2010;165: 438–455. 10.1016/j.ygcen.2009.04.017 19393655

[44] Weeg MS, Bass AH. Central lateral line pathways in a vocalizing fish. J Comp Neurol. 2000;418: 41–64. 10701755

[45] Matragrano LL, Beaulieu M, Phillip JO, Rae AI, Sanford SE, Sockman KW, et al Rapid effects of hearing song on catecholaminergic activity in the songbird auditory pathway. PLoS One. 2012;7: e39388 10.1371/journal.pone.0039388 22724011 PMC3378548

[46] Lynch KS, Diekamp B, Ball GF. Colocalization of immediate early genes in catecholamine cells after song exposure in female zebra finches (*Taeniopygia guttata*). Brain Behav Evol. 2012;79: 252–260. 10.1159/000337533 22572406 PMC3606879

[47] Chaube R, Joy KP. Brain tyrosine hydroxylase in the catfish Heteropneustes fossilis: annual and circadian variations, and sex and regional differences in enzyme activity and some kinetic properties. Gen Comp Endocrinol. 2003;130: 29–40. 12535622 10.1016/s0016-6480(02)00529-4

[48] Heimovics SA, Prior NH, Maddison CJ, Soma KK. Rapid and widespread effects of 17beta-estradiol on intracellular signaling in the male songbird brain: a seasonal comparison. Endocrinology. 2012;153: 1364–1376. 10.1210/en.2011-1525 22294743

[49] Smeets WJ, Gonzalez A. Catecholamine systems in the brain of vertebrates: new perspectives through a comparative approach. Brain Res Brain Res Rev. 2000;33: 308–379. 11011071 10.1016/s0165-0173(00)00034-5

[50] Dufour S, Sebert ME, Weltzien FA, Rousseau K, Pasqualini C. Neuroendocrine control by dopamine of teleost reproduction. J Fish Biol. 2010;76: 129–160. 10.1111/j.1095-8649.2009.02499.x 20738703

[51] Fontaine R, Affaticati P, Yamamoto K, Jolly C, Bureau C, Baloche S, et al Dopamine inhibits reproduction in female zebrafish (*Danio rerio*) via three pituitary D2 receptor subtypes. Endocrinology. 2013;154: 807–818. 10.1210/en.2012-1759 23295741

[52] Linard B, Anglade I, Corio M, Navas JM, Pakdel F, Saligaut C, et al Estrogen receptors are expressed in a subset of tyrosine hydroxylase-positive neurons of the anterior preoptic region in the rainbow trout. Neuroendocrinology. 1996;63: 156–165. 9053780 10.1159/000126952

[53] Fergus DJ, Bass AH. Localization and divergent profiles of estrogen receptors and aromatase in the vocal and auditory networks of a fish with alternative mating tactics. J Comp Neurol. 2013;521: 2850–2869. 10.1002/cne.23320 23460422 PMC3688646

[54] Forlano PM, Deitcher DL, Bass AH. Distribution of estrogen receptor alpha mRNA in the brain and inner ear of a vocal fish with comparisons to sites of aromatase expression. J Comp Neurol. 2005;483: 91–113. 15672394 10.1002/cne.20397

[55] Forlano PM, Marchaterre M, Deitcher DL, Bass AH. Distribution of androgen receptor mRNA expression in vocal, auditory, and neuroendocrine circuits in a teleost fish. J Comp Neurol. 2010;518: 493–512. 10.1002/cne.22233 20020540 PMC2976675

[56] Mamta SK, Raghuveer K, Sudhakumari CC, Rajakumar A, Basavaraju Y, Senthilkumaran B. Cloning and expression analysis of tyrosine hydroxylase and changes in catecholamine levels in brain during ontogeny and after sex steroid analogues exposure in the catfish, *Clarias batrachus* . Gen Comp Endocrinol. 2014;197: 18–25. 10.1016/j.ygcen.2013.11.022 24315863

[57] Weltzien FA, Pasqualini C, Sebert ME, Vidal B, Le Belle N, Kah O, et al Androgen-dependent stimulation of brain dopaminergic systems in the female European eel (*Anguilla anguilla*). Endocrinology. 2006;147: 2964–2973. 16543374 10.1210/en.2005-1477

[58] Forlano PM, Deitcher DL, Myers DA, Bass AH. Anatomical distribution and cellular basis for high levels of aromatase activity in the brain of teleost fish: aromatase enzyme and mRNA expression identify glia as source. J Neurosci. 2001;21: 8943–8955. 11698605 10.1523/JNEUROSCI.21-22-08943.2001 PMC6762278

[59] Maney DL, Bernard DJ, Ball GF. Gonadal steroid receptor mRNA in catecholaminergic nuclei of the canary brainstem. Neurosci Lett. 2001;311: 189–192. 11578826 10.1016/s0304-3940(01)02157-7

[60] Matragrano LL, LeBlanc MM, Chitrapu A, Blanton ZE, Maney DL. Testosterone alters genomic responses to song and monoaminergic innervation of auditory areas in a seasonally breeding songbird. Dev Neurobiol. 2013;73: 455–468. 10.1002/dneu.22072 23362219

[61] Forlano PM, Bass AH. Steroid regulation of brain aromatase expression in glia: Female preoptic and vocal motor nuclei. J Neurobiol. 2005;65: 50–58. 16010669 10.1002/neu.20178

[62] Forlano PM, Bass AH. Seasonal plasticity of brain aromatase mRNA expression in glia: Divergence across sex and vocal phenotypes. J Neurobiol. 2005;65: 37–49. 16003720 10.1002/neu.20179

[63] Forlano PM, Schlinger BA, Bass AH. Brain aromatase: new lessons from non-mammalian model systems. Front Neuroendocrinol. 2006;27: 247–274. 16828853 10.1016/j.yfrne.2006.05.002

[64] Callard GV, Tchoudakova AV, Kishida M, Wood E. Differential tissue distribution, developmental programming, estrogen regulation and promoter characteristics of cyp19 genes in teleost fish. J Steroid Biochem Mol Biol. 2001;79: 305–314. 11850237 10.1016/s0960-0760(01)00147-9

[65] Pawlisch BA, Kelm-Nelson CA, Stevenson SA, Riters LV. Behavioral indices of breeding readiness in female European starlings correlate with immunolabeling for catecholamine markers in brain areas involved in sexual motivation. Gen Comp Endocrinol. 2012;179: 359–368. 10.1016/j.ygcen.2012.09.007 22999823

[66] Falcon J, Besseau L, Sauzet S, Boeuf G. Melatonin effects on the hypothalamo-pituitary axis in fish. Trends Endocrinol Metab. 2007;18: 81–88. 17267239 10.1016/j.tem.2007.01.002

[67] Chaube R, Joy KP. Effects of altered photoperiod and temperature, serotonin-affecting drugs, and melatonin on brain tyrosine hydroxylase activity in female catfish, Heteropneustes fossilis: a study correlating ovarian activity changes. J Exp Zool. 2002;293: 585–593. 12410607 10.1002/jez.10185

[68] Sebert ME, Legros C, Weltzien FA, Malpaux B, Chemineau P, Dufour S. Melatonin activates brain dopaminergic systems in the eel with an inhibitory impact on reproductive function. J Neuroendocrinol. 2008;20: 917–929. 10.1111/j.1365-2826.2008.01744.x 18445127

[69] Mazurais D, Brierley I, Anglade I, Drew J, Randall C, Bromage N, et al Central melatonin receptors in the rainbow trout: comparative distribution of ligand binding and gene expression. J Comp Neurol. 1999;409: 313–324. 10379923 10.1002/(sici)1096-9861(19990628)409:2<313::aid-cne11>3.0.co;2-1

[70] El Halawani ME, Kang SW, Leclerc B, Kosonsiriluk S, Chaiseha Y. Dopamine-melatonin neurons in the avian hypothalamus and their role as photoperiodic clocks. Gen Comp Endocrinol. 2009;163: 123–127. 10.1016/j.ygcen.2008.11.030 19114045

[71] Kang SW, Leclerc B, Kosonsiriluk S, Mauro LJ, Iwasawa A, El Halawani ME. Melanopsin expression in dopamine-melatonin neurons of the premammillary nucleus of the hypothalamus and seasonal reproduction in birds. Neuroscience. 2010;170: 200–213. 10.1016/j.neuroscience.2010.06.082 20620198

[72] Uz T, Arslan AD, Kurtuncu M, Imbesi M, Akhisaroglu M, Dwivedi Y, et al The regional and cellular expression profile of the melatonin receptor MT1 in the central dopaminergic system. Brain Res Mol Brain Res. 2005;136: 45–53. 15893586 10.1016/j.molbrainres.2005.01.002

[73] Sabban EL, Maharjan S, Nostramo R, Serova LI. Divergent effects of estradiol on gene expression of catecholamine biosynthetic enzymes. Physiol Behav. 2010;99: 163–168. 10.1016/j.physbeh.2009.07.011 19638280

[74] Heimovics SA, Cornil CA, Ellis JM, Ball GF, Riters LV. Seasonal and individual variation in singing behavior correlates with alpha 2-noradrenergic receptor density in brain regions implicated in song, sexual, and social behavior. Neuroscience. 2011;182: 133–143. 10.1016/j.neuroscience.2011.03.012 21397668 PMC3085591

[75] Riters LV, Eens M, Pinxten R, Ball GF. Seasonal changes in the densities of alpha(2) noradrenergic receptors are inversely related to changes in testosterone and the volumes of song control nuclei in male European starlings. J Comp Neurol. 2002;444: 63–74. 11835182 10.1002/cne.10131

[76] d'Aldin C, Eybalin M, Puel JL, Charachon G, Ladrech S, Renard N, et al Synaptic connections and putative functions of the dopaminergic innervation of the guinea pig cochlea. Eur Arch Otorhinolaryngol. 1995;252: 270–274. 7576583 10.1007/BF00185388

[77] Darrow KN, Simons EJ, Dodds L, Liberman MC. Dopaminergic innervation of the mouse inner ear: evidence for a separate cytochemical group of cochlear efferent fibers. J Comp Neurol. 2006;498: 403–414. 16871528 10.1002/cne.21050 PMC1805779

[78] Eybalin M, Charachon G, Renard N. Dopaminergic lateral efferent innervation of the guinea-pig cochlea: immunoelectron microscopy of catecholamine-synthesizing enzymes and effect of 6-hydroxydopamine. Neuroscience. 1993;54: 133–142. 8100046 10.1016/0306-4522(93)90389-w

[79] Maison SF, Liu XP, Eatock RA, Sibley DR, Grandy DK, Liberman MC. Dopaminergic signaling in the cochlea: receptor expression patterns and deletion phenotypes. J Neurosci. 2012;32: 344–355. 10.1523/JNEUROSCI.4720-11.2012 22219295 PMC3313790

[80] Ruel J, Nouvian R, Gervais d'Aldin C, Pujol R, Eybalin M, Puel JL. Dopamine inhibition of auditory nerve activity in the adult mammalian cochlea. Eur J Neurosci. 2001;14: 977–986. 11595036 10.1046/j.0953-816x.2001.01721.x

[81] Ruel J, Wang J, Rebillard G, Eybalin M, Lloyd R, Pujol R, et al Physiology, pharmacology and plasticity at the inner hair cell synaptic complex. Hear Res. 2007;227: 19–27. 17079104 10.1016/j.heares.2006.08.017

[82] d'Aldin C, Puel JL, Leducq R, Crambes O, Eybalin M, Pujol R. Effects of a dopaminergic agonist in the guinea pig cochlea. Hear Res. 1995;90: 202–211. 8974998 10.1016/0378-5955(95)00167-5

[83] Gaborjan A, Lendvai B, Vizi ES. Neurochemical evidence of dopamine release by lateral olivocochlear efferents and its presynaptic modulation in guinea-pig cochlea. Neuroscience. 1999;90: 131–138. 10188940 10.1016/s0306-4522(98)00461-8

[84] Niu X, Canlon B. The signal transduction pathway for the dopamine D1 receptor in the guinea-pig cochlea. Neuroscience. 2006;137: 981–990. 16330149 10.1016/j.neuroscience.2005.10.044

[85] Garrett AR, Robertson D, Sellick PM, Mulders WH. The actions of dopamine receptors in the guinea pig cochlea. Audiol Neurootol. 2011;16: 145–157. 10.1159/000316674 20668375

[86] Furukawa T. Effects of efferent stimulation on the saccule of goldfish. J Physiol. 1981;315: 203–215. 7310707 10.1113/jphysiol.1981.sp013742 PMC1249377

[87] Edds-Walton PL, Holstein GR, Fay RR. Gamma-aminobutyric acid is a neurotransmitter in the auditory pathway of oyster toadfish, *Opsanus tau* . Hear Res. 2010;262: 45–55. 10.1016/j.heares.2010.01.008 20097279 PMC2878777

[88] Maison SF, Adams JC, Liberman MC. Olivocochlear innervation in the mouse: immunocytochemical maps, crossed versus uncrossed contributions, and transmitter colocalization. J Comp Neurol. 2003;455: 406–416. 12483691 10.1002/cne.10490 PMC1805785

[89] Boyle R, Rabbitt RD, Highstein SM. Efferent control of hair cell and afferent responses in the semicircular canals. J Neurophysiol. 2009;102: 1513–1525. 10.1152/jn.91367.2008 19571186 PMC2746798

[90] Filippi A, Mueller T, Driever W. vglut2 and gad expression reveal distinct patterns of dual GABAergic versus glutamatergic cotransmitter phenotypes of dopaminergic and noradrenergic neurons in the zebrafish brain. J Comp Neurol. 2014;522: 2019–2037. 10.1002/cne.23524 24374659 PMC4288968

[91] Drescher MJ, Cho WJ, Folbe AJ, Selvakumar D, Kewson DT, Abu-Hamdan MD, et al An adenylyl cyclase signaling pathway predicts direct dopaminergic input to vestibular hair cells. Neuroscience. 2010;171: 1054–1074. 10.1016/j.neuroscience.2010.09.051 20883745 PMC3025754

[92] Kapsimali M, Vidal B, Gonzalez A, Dufour S, Vernier P. Distribution of the mRNA encoding the four dopamine D(1) receptor subtypes in the brain of the european eel (*Anguilla anguilla*): comparative approach to the function of D(1) receptors in vertebrates. J Comp Neurol. 2000;419: 320–343. 10723008 10.1002/(sici)1096-9861(20000410)419:3<320::aid-cne5>3.0.co;2-f

[93] Pasqualini C, Weltzien FA, Vidal B, Baloche S, Rouget C, Gilles N, et al Two distinct dopamine D2 receptor genes in the European eel: molecular characterization, tissue-specific transcription, and regulation by sex steroids. Endocrinology. 2009;150: 1377–1392. 10.1210/en.2008-0578 18974275

[94] Khan KM, Hatfield JS, Drescher MJ, Drescher DG. The histochemical localization of acetylcholinesterase in the rainbow trout saccular macula by electron microscopy. Neurosci Lett. 1991;131: 109–112. 1791968 10.1016/0304-3940(91)90348-w

[95] Sugihara I. Efferent innervation in the goldfish saccule examined by acetylcholinesterase histochemistry. Hear Res. 2001;153: 91–99. 11223299 10.1016/s0378-5955(00)00259-8

[96] Koppl C. Evolution of the octavolateralis efferent system In: Ryugo DK, Fay RR, Popper AN, editors. Auditory and Vestibular Efferents. New York: Springer; 2011 pp. 217–259.

[97] Nakajima Y, Wang DW. Morphology of afferent and efferent synapses in hearing organ of the goldfish. J Comp Neurol. 1974;156: 403–416. 4414721 10.1002/cne.901560403

[98] Rabbitt RD, Brownell WE. Efferent modulation of hair cell function. Curr Opin Otolaryngol Head Neck Surg. 2011;19: 376–381. 10.1097/MOO.0b013e32834a5be1 22552698 PMC3343276

[99] Tomchik SM, Lu ZM. Modulation of auditory signal-to-noise ratios by efferent stimulation. Journal of Neurophysiology. 2006;95: 3562–3570. 16554519 10.1152/jn.00063.2006. PMC1693966

[100] McIver EL, Marchaterre MA, Rice AN, Bass AH. Novel underwater soundscape: acoustic repertoire of plainfin midshipman fish. J Exp Biol. 2014;217: 2377–2389. 10.1242/jeb.102772 24737759

[101] Lu Z, Fay RR. Acoustic response properties of single neurons in the central posterior nucleus of the thalamus of the goldfish, *Carassius auratus* . J Comp Physiol A. 1995;176: 747–760. 7776269 10.1007/BF00192623

[102] Sisneros JA. Adaptive hearing in the vocal plainfin midshipman fish: getting in tune for the breeding season and implications for acoustic communication. Integr Zool. 2009;4: 33–42. 10.1111/j.1749-4877.2008.00133.x 21392275

[103] Goebrecht GKE, Kowtoniuk RA, Kelly BG, Kittelberger JM. Sexually-dimorphic expression of tyrosine hydroxylase immunoreactivity in the brain of a vocal teleost fish (*Porichthys notatus*). J Chem Neuroanat. 2014;56: 13–34. 10.1016/j.jchemneu.2014.01.001 24418093

[104] Weeg MS, Bass AH. Frequency response properties of lateral line superficial neuromasts in a vocal fish, with evidence for acoustic sensitivity. J Neurophysiol. 2002;88: 1252–1262. 12205146 10.1152/jn.2002.88.3.1252

[105] Appeltants D, Del Negro C, Balthazart J. Noradrenergic control of auditory information processing in female canaries. Behav Brain Res. 2002;133: 221–235. 12110456 10.1016/s0166-4328(02)00005-0

[106] Lynch KS, Ball GF. Noradrenergic deficits alter processing of communication signals in female songbirds. Brain Behav Evol. 2008;72: 207–214. 10.1159/000157357 18815444

